# Mo‐O‐C Between MoS_2_ and Graphene Toward Accelerated Polysulfide Catalytic Conversion for Advanced Lithium‐Sulfur Batteries

**DOI:** 10.1002/advs.202201579

**Published:** 2022-06-05

**Authors:** Jiayu Zhang, Guobao Xu, Qi Zhang, Xue Li, Yi Yang, Liwen Yang, Jianyu Huang, Guangmin Zhou

**Affiliations:** ^1^ School of Materials Science and Engineering Xiangtan University Hunan 411105 China; ^2^ Shenzhen Geim Graphene Center Tsinghua–Berkeley Shenzhen Institute & Tsinghua Shenzhen International Graduate School Tsinghua University Shenzhen 518055 China; ^3^ School of Physics and Optoelectronics Xiangtan University Hunan 411105 China

**Keywords:** DFT calculation, hetero‐interface, in situ Raman spectrum, lithium‐sulfur batteries, separator modification

## Abstract

MoS_2_/C composites constructed with van der Waals forces have been extensively applied in lithium–sulfur (Li–S) batteries. However, the catalytic conversion effect on polysulfides is limited because the weak electronic interactions between the composite interfaces cannot fundamentally improve the intrinsic electronic conductivity of MoS_2_. Herein, density functional theory calculations reveal that the MoS_2_ and nitrogen‐doped carbon composite with an Mo–O–C bond can promote the catalytic conversion of polysulfides with a Gibbs free energy of only 0.19 eV and a low dissociation energy barrier of 0.48 eV, owing to the strong covalent coupling effect on the heterogeneous interface. Guided by theoretical calculations, a robust MoS_2_ strongly coupled with a 3D carbon matrix composed of nitrogen‐doped reduced graphene oxide and carbonized melamine foam is designed and constructed as a multifunctional coating layer for lithium–sulfur batteries. As a result, excellent electrochemical performance is achieved for Li–S batteries, with a capacity of 615 mAh g^–1^ at 5 C, an areal capacity of 6.11 mAh cm^–2^, and a low self‐discharge of only 8.6% by resting for five days at 0.5 C. This study opens a new avenue for designing 2D material composites toward promoted catalytic conversion of polysulfides.

## Graphical abstract

Density functional theory calculations reveal that the MoS_2_/C composite with the strong covalent coupling effect can promote the catalytic conversion of polysulfides due to a low Gibbs free energy and dissociation energy barrier. Subsequently, MoS_2_/C with Mo–O–C bond is constructed and possesses strong lithium polysulfide chemical adsorption and abundant catalytic sites, enabling improved electrochemical performance of lithium–sulfur batteries.

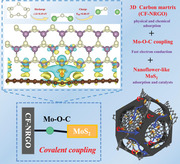

## 1 Introduction

In view of the rapid development of portable electronics and electric vehicles, there is an urgent need to develop high‐energy‐density and low‐cost energy storage systems. Lithium–sulfur (Li–S) batteries are among the most promising next‐generation battery systems because of their high theoretical capacity (1675 mAh g^–1^), high theoretical energy density (2600 Wh kg^–1^),^[^ ^1^ ^]^ and low cost (approximately 150 $ ton^–1^ of sulfur).^[^ ^2^ ^]^ Nevertheless, the commercialization of Li‐S batteries is limited by several challenges, such as the low utilization of sulfur, large volume expansion (≈80%),^[^ ^3^ ^]^ and “shuttle effect” caused by soluble lithium polysulfides (LiPSs), leading to rapid capacity fading, severe self‐discharge, low Coulombic efficiency, and poor cycling stability.^[^ ^4^ ^]^

Based on theoretical calculations and experimental research, tremendous efforts have been made to mitigate the issues caused by the shuttle effect, including the construction of various nano‐structured sulfur host materials,^[^ ^5^, ^6^ ^]^ the design of advanced electrolytes,^[^ ^7^ ^]^ and the use of multifunctional separators.^[^ ^8^, ^9^ ^]^ Among these, functional separators have been considered a significant and efficient approach to impede the diffusion of LiPSs and improve the electrochemical performance of Li–S batteries. Owing to their high conductivity, large specific surface area, and lightweight,^[^ ^10^, ^11^, ^12^ ^]^ various types of carbonaceous materials have been used to mitigate the shuttle effect of LiPSs. Nevertheless, nonpolar carbon materials are usually limited in alleviating the shuttle effect of LiPSs because of their weak interactions with polar LiPSs.^[^ ^13^ ^]^ Therefore, several polar materials, such as, transitional metal oxides^[^ ^14^, ^15^, ^16^ ^]^ and sulfides^[^ ^17^, ^18^, ^19^ ^]^ which can adsorb LiPSs owing to their strong chemical interactions, have been applied as coated functional materials on separators. In particular, MoS_2_ materials have been shown to promote the catalytic conversion of LiPSs.^[^ ^20^ ^]^ Nonetheless, it is difficult to achieve highly effective bidirectional solid–liquid–solid conversion of sulfur–LiPSs–lithium sulfides with individual components. As a result, various composites have been used to address these issues. On the one hand, the construction of MoS_2_, to increase the exposure of active edge sites, combined with carbon materials is designed to boost the trapping and conversion of LiPSs in Li–S batteries.^[^ ^21^, ^22^ ^]^ On the other hand, researcher has focused on the micro/nanostructures of MoS_2_/C composites via van der Waals forces and utilize their synergistic effect to enhance the electrochemical performance of Li–S batteries.^[^ ^23^, ^24^, ^25^ ^]^ Although these MoS_2_/C composites can effectively suppress the shuttle effect of LiPSs, they are mainly bound through van der Waals bonds, and it is difficult to effectively exert the synergistic effect of the two components owing to the weak electronic pathway of the heterogeneous interface. Hence, constructing the MoS_2_/C composites with strongly coupled valence bonds and studying the intrinsic mechanism of LiPSs inhibition are important for the optimization of Li–S batteries.

In this study, we verified that the intrinsic electronic conductivity of MoS_2_ and the smooth electronic pathway of the heterogeneous interface of MoS_2_/C can significantly promote the transformation of LiPSs and decomposition of Li_2_S, respectively. An MoS_2_/C composite was constructed using robust MoS_2_ with abundant active sites covalently coupled with a 3D carbon matrix composed of nitrogen‐doped reduced graphene oxide and carbonized melamine foam (MoS_2_@CF‐NRGO). In this scheme, the strong interfacial connection of MoS_2_@CF‐NRGO ensured ultrafast electronic transfer and structural stability between MoS_2_ and the 3D carbon matrix. Because the composite possessed strong LiPSs chemical adsorption, rapid electronic conduction ability, and abundant catalytic sites, Li–S batteries with the MoS_2_@CF‐NRGO coated separator delivered excellent rate capability, cycling stability, favorable anti‐self‐discharge capacity, and high/low‐temperature performance. Even for an Li–S battery with high‐areal‐capacity sulfur loading (8.0 mg cm^–2^), a reversible areal capacity of 5.58 mAh cm^–2^ was still achieved after 50 cycles at 0.2 C with 91.3% capacity retention. More importantly, based on density functional theory (DFT) calculations and in situ Raman spectrum analyses, we found that the excellent performance originated from the pivotal role of MoS_2_@CF‐NRGO in the rapid anchoring‐diffusion transformation of LiPSs.

## 2 Results and Discussion

### 2.1 Theory‐Oriented Design

Combining theoretical calculations with experimental studies, we aimed to reveal the relationship between the intrinsic physicochemical properties of MoS_2_ and the anchoring‐diffusion transformation of LiPSs, explore the influence of the electron transport properties of the interface of MoS_2_ based composites on the dissociation kinetics of Li_2_S, and propose a well‐developed strategy for the MoS_2_/C composite toward promoted Li–S chemistry.

**Figure 1:**
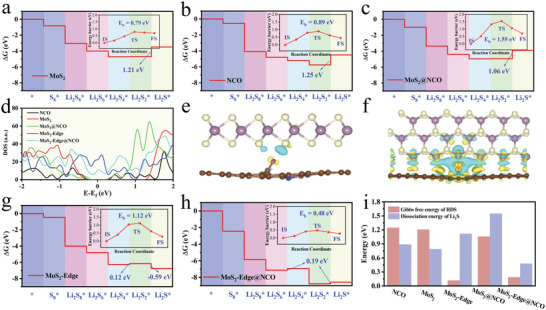
Energy profiles for the reduction of LiPSs on a) pure MoS_2_, b) NCO, c) MoS_2_@NCO, g) MoS_2_‐Edge, and h) MoS_2_‐Edge@NCO substrates. (Insets) The energy profiles of Li_2_S decomposition on corresponding substrates. d) Density of state (DOS) calculations for different models. Optimized geometries of electron distribution at the heterointerface of e) MoS_2_@NCO and f) MoS_2_‐Edge@NCO; yellow areas: charge accumulation; blue areas: charge depletion. The iso‐surface is set to 0.002 eV Å^–3^. i) The Gibbs free energy of the reduction of LiPSs and dissociation energy of Li_2_S for different models.

First‐principles calculations were performed based on DFT implemented by the Vienna Ab‐initio Simulation Package presented in **Figure** **1** and Figure S1–S5, Supporting Information. As shown in Figure 1a, pure MoS_2_ exhibited poor catalytic activity for the transformation of LiPSs owing to the slow reaction kinetics of the Li_2_S_2_*→Li_2_S* process with a high Gibbs free energy of 1.21 eV. However, pure MoS_2_ promoted the dissociation of Li_2_S, with a relatively low dissociation energy barrier of 0.79 eV. After pure MoS_2_ was coupled to nitrogen‐doped carbon with oxygen‐containing functional groups (NCO), the composite exhibited a lower Gibbs free energy (Figure 1c) than pure MoS_2_ and the NCO substrate (Figure 1b). Density of state (DOS) calculations indicated the enhanced catalytic activity of MoS_2_@NCO for the transformation of LiPSs originating from the enhanced electronic conductivity. As shown in Figure 1d, the energy gap of MoS_2_@NCO was narrower than that of pure MoS_2_. However, the dissociation energy barrier of Li_2_S increased, limiting the charging process as a result of the electrons being localized at the O atom on the interface of MoS_2_@NCO (Figure 1e), blocking the electron transport channel between the interface layers.

Therefore, an ideal MoS_2_/C composite for Li–S batteries could be constructed from two aspects: enhancing the electronic conductivity of MoS_2_ and ensuring a smooth electronic pathway of the heterogeneous interface. The DOS analysis in Figure 1d indicates that the MoS_2_ nanoribbon exposed numerous molybdenum atoms possessing a metallic nature because the energy band gap was zero. In addition, the electrons were concentrated near the Fermi level, which is beneficial for the anchoring and transformation of LiPSs. As shown in Figure 1g, the Gibbs free energy of the rate‐determining step was only 0.12 eV, indicating an excellent catalytic activity of the MoS_2_ nanoribbon for the transformation of the LiPSs. However, the dissociation of Li_2_S on the MoS_2_ nanoribbon was difficult because of the high dissociation energy barrier of Li_2_S (1.12 eV). As shown in Figure 1f, after coupling the MoS_2_ nanoribbon with NCO via the Mo–O bond (MoS_2_‐Edge@NCO), the composite enabled electrons to conduct smoothly from the MoS_2_ nanoribbon to NCO, leading to an electron‐deficient MoS_2_ surface, accelerating the dissociation kinetics of Li_2_S. As shown in Figure 1h, the MoS_2_‐Edge@NCO could not only easily catalyze the polysulfide conversion with a Gibbs free energy of only 0.19 eV, but also promote the dissociation of Li_2_S with a low dissociation energy barrier of 0.48 eV compared with other calculated samples shown in Figure 1i.

### 2.2 Synthesis and Characterization

**Figure 2:**
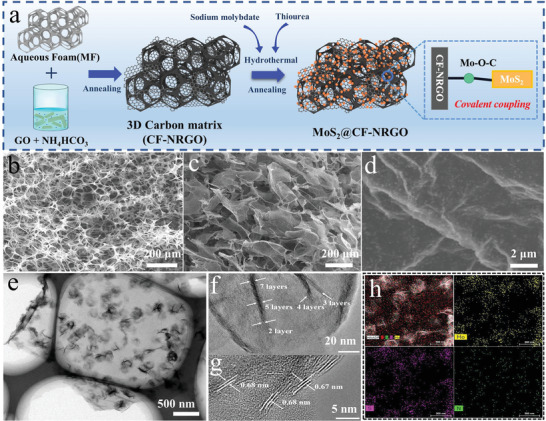
a) Schematic illustration of the preparation stages for MoS_2_@CF‐NRGO. SEM images of b) carbonized MF and c,d) MoS_2_@CF‐NRGO. e) Transmission electron microscopy (TEM) and f,g) HR‐TEM images of MoS_2_@CF‐NRGO. h) EDS mapping images of MoS_2_@CF‐NRGO and the corresponding images of Mo, S, and N.

Based on the above computational results, we designed the MoS_2_@CF‐NRGO composite and applied it to Li–S batteries as a modified layer of a polypropylene separator (MoS_2_@CF‐NRGO/PP) (**Figure** **2**a). First, GO was dispersed in a solution containing NH_4_HCO_3_ to obtain a GO/NH_4_^+^ composite via electrostatic self‐assembly. Subsequently, melamine foam (MF) was immersed in the above solution to prepare the MF‐GO/NH_4_^+^ composite. After annealing, a 3D carbon matrix composed of CF‐NRGO was fabricated. Second, the obtained 3D carbon matrix was added to a mixed solution of thiourea and sodium molybdate, and treated in a hydrothermal reaction with further calcination to acquire 3D porous MoS_2_@CF‐NRGO. MoS_2_ covalent coupling on the 3D carbon matrix via the Mo–O–C bond enhances the electronic conductivity between MoS_2_ and the 3D carbon matrix, thus improving the capability of MoS_2_ to catalyze LiPSs and dissociate Li_2_S. The morphologies of the samples were characterized using scanning electron microscopy (SEM), transmission electron microscopy (TEM), and high‐resolution TEM. As depicted in Figure 2b, CF with smooth skeletons exhibited a 3D framework architecture, which could be used as an accommodation or support system. After annealing the MF‐GO/NH_4_^+^, the CF‐NRGO was fabricated and inherited the 3D framework architecture of CF (Figure S6a—c, Supporting Information), in which the NRGO with continuous and wrinkled nanosheet structures was uniformly dispersed in CF, providing numerous active sites for the uniform growth of MoS_2_. Figure 2c,d shows the SEM images of MoS_2_@CF‐NRGO at various magnifications. The composite maintained a 3D carbon matrix structure, which provided favorable conditions for Li^+^ shuttling and increased the effective infiltration of LiPSs. Moreover, the surface of the NRGO exhibited uniform growth of MoS_2_ without agglomeration, enabling the hybrids to possess more edge sites of MoS_2_, thus increasing the adsorption ability and catalytic activity of LiPSs. To further observe the detailed structures of MoS_2_@CF‐NRGO, Figure 2e and Figure S7, Supporting Information demonstrate that the MoS_2_ with nanoflower‐like morphology was composed of several lamellar nanosheets and strongly coupled on the surface of the NRGO. These results indicate that MoS_2_@CF‐NRGO provides a large number of adsorption and catalytic sites for the LiPSs conversion reaction and that internal synergetic effects increase the anchoring‐diffusion transformation process of LiPSs. In addition, the interlayer spacing of MoS_2_ was approximately 0.68 nm, corresponding to the (002) plane of MoS_2_ (Figure 2f,g). Meanwhile, elemental mapping (Figure 2h) not only further revealed the existence of Mo and S, but also confirmed the homogeneous distribution of N on the NRGO.

**Figure 3:**
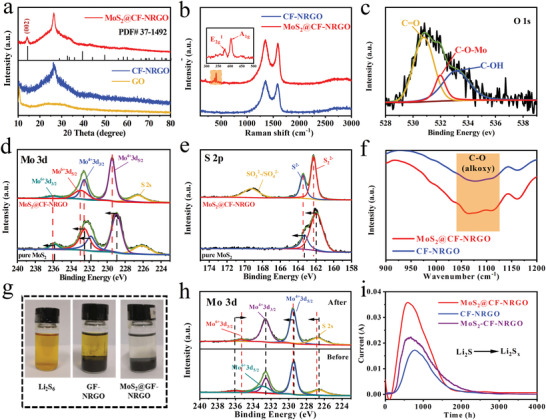
a) X‐ray diffraction (XRD) patterns and b) Raman spectra of MoS_2_@CF‐NRGO, CF‐NRGO and GO. c) XPS spectra for O 1s of the MoS_2_@CF‐NRGO, and the comparison of d) Mo 3d, and e) S 2p of the MoS_2_@CF‐NRGO and MoS_2_@hydrothermal. f) FTIR spectra of the MoS_2_@CF‐NRGO and CF‐NRGO. g) Polysulfide adsorption test for MoS_2_@CF‐NRGO and CF‐NRGO and h) X‐ray photoelectron spectroscopy (XPS) spectra of Mo 3d of the MoS_2_@CF‐NRGO before and after adsorption with Li_2_S_6_. i) Charge curves at 2.4 V of a Li_2_S_8_/tetraglyme solution on different the surfaces of MoS_2_@CF‐NRGO, CF‐NRGO, and MoS_2_‐CF‐NRGO electrodes.

The crystal phase compositions of the as‐prepared samples were identified using X‐ray diffraction (XRD) (**Figure** **3**a). From the CF‐NRGO pattern, one strong diffraction peak at approximately 26° was observed, suggesting that GO had been reduced. However, there are residual oxygen‐containing functional groups on RGO, as shown in the Fourier transform infrared spectroscopy (FTIR) pattern in Figure S8, Supporting Information,^[^ ^26^ ^]^ which are beneficial for the nucleation and growth of MoS_2_. As a comparison, the characteristic diffraction peak of the graphitic structure in MoS_2_@CF‐NRGO shifted slightly to a higher diffraction angle, indicating a slight increase in lattice distortion. This may be derived from the strong interaction between MoS_2_ and CF‐NRGO, which can be further proved by the XRD peaks of CF after hydrothermal treatment (Figure S9, Supporting Information). In addition, the characteristic diffraction peak corresponding to the (002) plane of MoS_2_ located at 13° was detected within MoS_2_@CF‐NRGO, attributed to the growth of MoS_2_ along the (002) crystal plane. Figure 3b displays the Raman spectra of the samples, where two typical peaks are denoted as graphitic carbon (G, at 1600 cm^–1^) and disordered carbon (D, at 1348 cm^–1^).^[^ ^27^ ^]^ Compared to the peak intensity ratio (*I*_D/G_ = 1.00) of CF‐NRGO, the ratio of MoS_2_@CF‐NRGO increased to 1.09, implying strong electronic coupling between MoS_2_ and CF‐NRGO, which is consistent with the XRD results. In addition, MoS_2_@CF‐NRGO exhibited two Raman bands at 380 and 406 cm^–1^, corresponding to the ^1^ *E*_2g_ (in‐plane optical vibration of the Mo–S bond in opposite directions) and *A*_1g_ (out‐of‐plane optical vibration of S atoms) active modes, respectively.^[^ ^28^ ^]^ Most importantly, the intensity value of *A*_1g_ to ^1^ *E*_2g_ in MoS_2_@CF‐NRGO (2.02) was significantly higher than that in MoS_2_ (1.44), as shown in Figure S10b, Supporting Information, demonstrating the presence of more active edge sites of MoS_2_ in MoS_2_@CF‐NRGO.^[^ ^29^ ^]^

X‐ray photoelectron spectroscopy (XPS) analysis of MoS_2_@CF‐NRGO was performed to characterize the chemical bonding states. O 1s spectrum was deconvoluted into three peaks centered at 530.78, 531.98, and 533.12 eV (Figure 3c) for MoS_2_@CF‐NRGO, which can be associated with C≐O, C–O—Mo, and C–OH, respectively^[^ ^30^ ^]^ demonstrating the formation of C–O–Mo bonds in MoS_2_@CF‐NRGO. Moreover, the Mo 3d spectrum of MoS_2_@CF‐NRGO (Figure 3d) exhibited five fitted characteristic peaks at 232.5 eV (Mo 3d_3/2_ of Mo^4+^), 229.4 eV (Mo 3d_5/2_ of Mo^4+^), 235.9 eV (Mo 3d_3/2_ of Mo^6+^), 233 eV (Mo 3d_5/2_ of Mo^6+^), and 226.6 eV (assigned to the S 2s of divalent sulfide ions),^[^ ^31^ ^]^ and the S 2s spectrum of MoS_2_@CF‐NRGO (Figure 3e) presented three fitted characteristic peaks located at 169.2, 163.4, and 162.2 eV, corresponding to SO_3_^2–^/SO_4_^2–^, S^2‐,^ and S_2_^2–^, respectively. Compared to the high‐resolution Mo 3d and S 2s spectra of pure MoS_2_, the binding energies of MoS_2_@CF‐NRGO shifted toward high‐energy direction, indicating a change in the electronic structure and electron density loss from MoS_2_.^[^ ^32^ ^]^ This is consistent with the results of the theoretical calculations. Meanwhile, the FTIR spectra showed that the ratio of C‐O in MoS_2_@CF‐NRGO was larger than that in CF‐NRGO (Figure 3f), further implying the formation C–O–Mo bonds in MoS_2_@CF‐NRGO.^[^ ^33^ ^]^ Subsequently, a 23.3% MoS_2_ content was determined in MoS_2_@CF‐NRGO using thermogravimetric analysis (Figure S11a, Supporting Information). The specific surface area and permanent porosity were examined using N_2_ adsorption/desorption (Figure S11b, Supporting Information). Although the specific surface area of MoS_2_@CF‐NRGO was smaller than that of CF‐NRGO owing to the introduction of MoS_2_, the diameter of the holes was concentrated in the range of less than 10 nm. Therefore, MoS_2_@CF‐NRGO had sufficient sites for the adsorption and catalytic conversion of LiPSs but did not affect Li^+^ transport.

### 2.3 Polysulfide Adsorption and Catalytic Conversion on MoS_2_@CF‐NRGO

A visual adsorption experiment was conducted as shown in Figure 3g. After 24 h, the initial yellowish Li_2_S_6_ solution became transparent in the presence of MoS_2_@CF‐NRGO, whereas the yellow color of the Li_2_S_6_ solution remained for CF‐NRGO. Moreover, the adsorbed Li_2_S_6_ solution was analyzed by UV absorption spectroscopy (Figure S12, Supporting Information), in which the characteristic peaks corresponding to S_6_^2−^ at approximately 260 nm presented a more obvious decrease in intensity in the spectra of MoS_2_@CF‐NRGO,^[^ ^34^ ^]^ suggesting strong chemical absorption of MoS_2_@CF‐NRGO toward LiPSs. XPS was further used to characterize MoS_2_@CF‐NRGO after the adsorption experiment. First, the XPS full spectrum of MoS_2_@CF‐rGO (Figure S13, Supporting Information) confirmed the co‐existence of Mo, S, C, N, and O. The characteristic peaks of Mo^6+^ decreased after the adsorption experiment (Figure 3h), indicating a strong chemical reaction between Mo^6+^ and Li_2_S_6_. Meanwhile, the positions of several Mo 3d peaks and the S 2p peaks exhibited a slight shift (Figure 3h and Figure S14a, Supporting Information), confirming the strong electronic interaction between MoS_2_ and LiPSs. However, in the C 1s spectrum with three fitted peaks (C–C (284.7 eV), C–N (286.3 eV), and O–C≐O (288.9 eV)),^[^ ^35^ ^]^ only the C–N peak changed, while the other peaks did not change before or after the adsorption experiment (Figure S14b, Supporting Information), indicating that the doping of nitrogen atoms could increase the adsorption ability of the carbon substrate to Li_2_S_6._

Furthermore, cyclic voltammetry (CV) of symmetric cells was conducted in a potential window from −1.5 to 1.5 V at a scan rate of 10 mV s^−1^ to evaluate the LiPSs catalytic ability of modified materials (Figure S15, Supporting Information). Compared with CF‐NRGO and MoS_2_‐CF‐NRGO (physical mixture of pure MoS_2_ and CF‐NRGO), the CV curve of MoS_2_@CF‐NRGO displayed a larger redox current, in which peaks C and D in the cathodic scan were assigned to the reduction of elemental sulfur to Li_2_S_6_ and Li_2_S_6_ to Li_2_S_2_/Li_2_S, respectively, and peaks A and B in the subsequent anodic scan represented opposite oxidation processes.^[^ ^36^ ^]^ In addition, Li_2_S precipitation and oxidation measurements were conducted to further reveal the preponderance of MoS_2_@CF‐NRGO in polysulfide conversion, as shown in Figure 3i and Figure S16, Supporting Information. Compared with the results of CF‐NRGO and MoS_2_‐CF‐NRGO, the responsivity of Li_2_S uncleation in MoS_2_@CF‐NRGO was more rapid, and the capacity of Li_2_S precipitation in MoS_2_@CF‐NRGO was higher (Figure S16, Supporting Information). More importantly, the oxidation of solid Li_2_S in MoS_2_@CF‐NRGO exhibited a more obvious enhancement than those in CF‐NRGO and MoS_2_‐CF‐NRGO (Figure 3i), suggesting a significantly reduced oxidation overpotential for Li_2_S conversion,^[^ ^37^ ^]^ which is consistent with the results of the theoretical calculations. The above results reveal that the synergistic effects of CF‐NRGO and MoS_2_ can significantly accelerate the transformation kinetics of LiPSs.

### 2.4 Electrochemical Performance of Li–S Batteries Constructed Using MoS_2_@CF‐NRGO

Before being applied to Li–S batteries, MoS_2_@CF‐NRGO/PP was investigated and analyzed. As shown in Figure S17a, Supporting Information, there was no appreciable delamination or deformation of MoS_2_@CF‐NRGO/PP after folding and bending, suggesting excellent flexibility and firm adhesion. A cross‐sectional SEM image shows an approximately 10 µm thick coating layer with an areal density of 0.3 mg cm^−2^ (Figures S17b and S18, Supporting Information). The SEM images of the samples (Figure S17c,d,e, Supporting Information) show that PP Celgard possessed a macroporous structure (>200 nm) (Figure S17c, Supporting Information), which is larger than the size of soluble LiPSs (<2 nm), leading to a serious shuttle effect on the LiPSs. The coating layer of CF‐NRGO and MoS_2_@CF‐NRGO was relatively fluffy, which provided favorable conditions for Li^+^ shuttling and physically blocked the shuttling of LiPSs. The above separators were then used to perform a permeation experiment a H‐type device (Figure S19, Supporting Information). Compared to PP and CF‐NRGO/PP, MoS_2_@CF‐rGO/PP exhibited the most effective ability to alleviate the shuttling of LiPSs. Furthermore, Li–Li symmetrical cells with different separators were used as shown in Figure S20, Supporting Information. The Li–Li symmetrical cell with MoS_2_@CF‐NRGO/PP showed a lower and more stable overpotential after 300 h than those of Li–Li symmetrical cells with PP and CF‐NRGO/PP, also proving favorable conditions for Li^+^ transfer and inhibition of lithium dendrite growth in MoS_2_@CF‐NRGO/PP.

**Figure 4:**
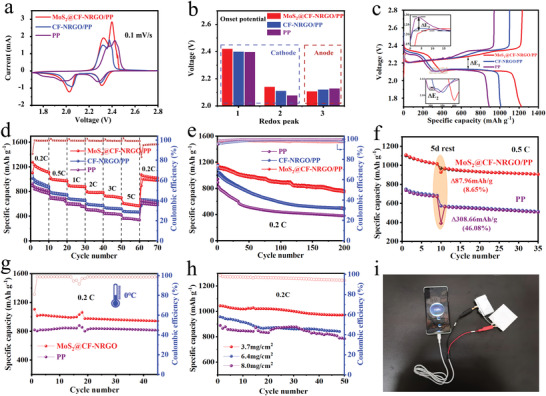
a) Cyclic voltammetry (CV) curves at a scan rate of 0.1 mV s^−1^, and b) the onset potentials for the electrochemical reaction stages of different separators. c) The first charge/discharge profiles at 0.2 C (inset: the corresponding enlarged part). d) Rate capacity and e) cycling capabilities at 0.2 C of a cell with the MoS_2_@CF‐NRGO/PP separator, CF‐NRGO/PP, and PP separators. f) Cycling performance at 0.5 C before and after rest of the MoS_2_@CF‐NRGO/PP and PP separators. g) Cycling performance at 0.2 C of different separators under 0 ℃. h) Cycling performance of the MoS_2_@CF‐NRGO/PP separator with high sulfur content. i) Soft‐pack cells charge a mobile phone, even in the folded state.

Subsequently, CV curves with different Li–S battery separators were first constructed within the potential range 1.7–2.8 V at a scan rate of 0.1 mV s^–1^ (**Figure** **4**a). Two clear cathodic peaks located at approximately 2.3 and 2.0 V, corresponding to the reduction from cyclic S_8_ to soluble lithium polysulfide (Li_2_S_n_, 4 ≤ *n* ≤ 8) and further conversion to Li_2_S_2_/Li_2_S, and two anode peaks at near 2.3 and 2.4 V can be ascribed to the delithiation of Li_2_S/Li_2_S_2_ and Li_2_S_n_. Moreover, compared to the CV curves of the contrastive samples, CV profile of MoS_2_@CF‐NRGO configuration delivered a higher peak current and smaller polarization voltage (Figure S22, Supporting Information), indicating the enhanced transformation of S_8_ to soluble LiPSs and then further to insoluble products (Li_2_S) and facilitated the oxidation of Li_2_S to sulfur. In addition, the onset potential values of all redox peaks, which were determined at a current density of 10 µA cm^–2^ beyond the baseline voltage as shown in Figure S23, Supporting Information,^[^ ^38^ ^]^ were measured based on different CV profiles (Figure 4b and Figure S24, Supporting Information). The MoS_2_@CF‐NRGO configuration showed the largest reduction peak and the smallest oxidation peak, indicating the preferable catalytic activity of MoS_2_@CF‐NRGO.^[^ ^39^ ^]^ The galvanostatic charge/discharge curves of all the samples presented one charge plateau and two discharge plateaus, which is consistent with the CV analysis (Figure 4c). The MoS_2_@CF‐NRGO configuration exhibited a the longer and more stable discharge profile with a significantly smaller polarization potential, ΔE_1_, further demonstrating the electrocatalytic effect of the heterostructure.^[^ ^40^ ^]^ Meanwhile, the MoS_2_@CF‐NRGO configuration exhibited the lowest overpotential Δ*E*_2_ and Δ*E*_3_ at the second discharge plateau and the first charge plateau, respectively, indicating the rapid conversion of soluble Li_2_S_4_ to insoluble Li_2_S_2_/Li_2_S and the transformation of soluble Li_2_S to sulfur, respectively, which is consistent with the theoretical calculations analysis. As expected, the MoS_2_@CF‐NRGO configuration delivered outstanding rate capacities of 1274.9, 1016.9, 901.4, 801.6, 731.7, and 615.1 mAh g^–1^, at 0.2, 0.5, 1, 2, 3, and 5 C, respectively, which were better than those of the CF‐NRGO configuration (1012.9, 782.2, 684.2, 599, 538.1, and 481.7 mAh g^–1^) and PP configuration (937.2, 721.7, 624.9, 526.2, 462.5, and 379.6 mAh g^–1^) (Figure 4d). The cycling performance of the MoS_2_@CF‐NRGO configuration was also measured at 0.2 C as shown in Figure 4e. The initial reversible discharge capacity was 1120 mAh g^–1^, and present an excellent capacity retention of 742.9 mAh g^–1^ was presented after 200 cycles with 99.6% average Coulombic efficiency. In comparison, the discharge capacities of the CF‐NRGO configuration and PP configuration rapidly faded from 1103.5 to 485.6 mAh g^−1^ and from 890.3 to 381 mAh g^−1^, respectively, after 200 cycles. Meanwhile, the galvanostatic charge/discharge profiles of Li‐S batteries with different separators for the 1st, 50th, 100th, 150th, and 200th cycles at 0.2 C exhibited the typical characteristics of two plateaus (Figure S25, Supporting Information). However, the charge/discharge voltage profiles of the MoS_2_@CF‐NRGO configuration were flatter profile than those of the others, demonstrating the excellent electrocatalytic ability of MoS_2_@CF‐NRGO. The MoS_2_@CF‐NRGO configuration also exhibited stable performance, even at a low electrolyte/sulfur ratio of 10 µL mg^–1^ (Figure S26, Supporting Information). The long‐term cycling stability was also tested at 1 C, as shown in Figure S27, Supporting Information. The MoS_2_@CF‐NRGO configuration exhibited an average decay rate of only 0.06% per cycle after 1000 cycles, which is significantly lower than those in the CF‐NRGO configuration (0.09%) and PP configuration (0.13% after 443 cycles). The electrochemical performance of the MoS_2_@CF‐NRGO configuration was comparable and even superior to that of reported compounds for Li–S batteries (Table S1).

To further understand the improved electrochemical performance of MoS_2_@CF‐NRGO configuration, electrochemical impedance spectroscopy (EIS) measurements and SEM characterization were performed on the cells before and after cycling. Figure S28a, Supporting Information presents Nyquist plots before cycling of the different configurations, in which the MoS_2_@CF‐NRGO configuration shows the smallest semicircle in the high‐frequency region, indicating the lowest *R*_ct_ and best electrolyte wettability. After 100 cycles, an additional semicircle was appeared in the Nyquist plots of all configurations (Figure S28b semicircle), which was attributed to the formed solid electrode/electrolyte interface.^[^ ^41^ ^]^ Compared with the contrastive samples, the MoS_2_@CF‐NRGO configuration showed the lowest *R*_ct_, electrode/electrolyte interface resistance, and Warburg impedance, demonstrating accelerated catalytic conversion of LiPSs and facilitated Li^+^ transport during cycling.^[^ ^42^ ^]^ Furthermore, the Li^+^ diffusion coefficient, D (cm^2^ s^–1^), of the MoS_2_@CF‐NRGO configuration before and after cycling, calculated from the EIS curves (Figure S29, Supporting Information), was higher than those of the PP and CF‐NRGO configurations (Figure S28c, Supporting Information). Li–S batteries with different separators were disassembled after 200 cycles to observe the curbing effect of multifunctional layer for LiPSs. Digital photographs and the corresponding SEM images are shown in Figure S30, Supporting Information. The clear yellow substance adhered to the surface of the PP separator due to the high concentration of insoluble Li_2_S_2_/Li_2_S in the electrolyte, whereas the surface of MoS_2_@CF‐NRGO/PP hardly exhibited a yellow substance (Figure S30a1,b1,c1, Supporting Information). Meanwhile, compared with the contrastive samples (Figure S30, Supporting Information), the surface of the lithium anode in the MoS_2_@CF‐NRGO configuration was the smoothest because it had the smallest deposition of Li_2_S_2_/Li_2_S, indicating the favorable catalytic conversion of LiPSs in MoS_2_@CF‐NRGO.

The suppressed shuttle effect of LiPSs and enhanced redox reaction dynamics endowed the MoS_2_@CF‐NRGO configuration with Mo–O–C bonds possessed excellent performance. Thus, several properties were further investigated from a practical perspective. First, the self‐discharge behavior of Li–S batteries was studied by resting for five days after 10 charge/discharge cycles at 0.5 C. As shown in Figure 4f, the discharge capacity of the MoS_2_@CF‐NRGO configuration after resting for five days is decreased slightly from 1015.8 to 927.9 mAh g^−1^, in which the average self‐discharge value was calculated to be only 8.6%. In contrast, the discharge capacity of the PP configuration is sharply decreased from 669.7 to 391.1 mAh g^−1^ and the self‐discharge value is reached to 46.08%. This result indicates that the MoS_2_@CF‐NRGO configuration can effectively relieve self‐discharge by suppressing the shuttle effect of LiPSs. From the galvanostatic charge/discharge curves shown in Figure S31, Supporting Information, the reversibility of the high‐voltage plateau was inferior for the PP configuration, in which the Q_H_ (capacity of the high plateau) retention was only 79.16% after rest. In contrast, the MoS_2_@CF‐NRGO configuration had a 97.25% retention rate after rest, which demonstrates its excellent LiPSs capture capability during the self‐discharge process.^[^ ^43^ ^]^ The self‐discharge value of the MoS_2_@CF‐NRGO configuration was also comparable to those reported for coating separators or host materials (Table S2, Supporting Information). In addition, the cycling performance was evaluated at high and low temperatures, as shown in Figure 4g and Figure S32, Supporting Information. Both configurations delivered an increased initial capacity at 60 ℃ and 0.2 C because of the facilitated diffusion of Li ions and electrochemical reactions at high temperatures (Figure S32, Supporting Information). Importantly, the MoS_2_@CF‐NRGO configuration still retained a relatively stable Coulombic efficiency because the barrier and reuse effect of the modified layer for LiPSs becomes more important at high temperatures.^[^ ^44^ ^]^ Moreover, the MoS_2_@CF‐NRGO configuration provided a higher capacity than the PP configuration with 0 ℃ at 0.2 C (Figure 4g), suggesting an enhanced catalytic effect in MoS_2_@CF‐NRGO. Furthermore, the cycling performance of the MoS_2_@CF‐NRGO configuration with various areal sulfur loading at 0.2 C was also investigated, as shown in Figure 4h. The areal capacities with 3.7, 6.4, and 8.0 mg cm^–2^ were 3.41, 5.30, and 6.11 mAh cm^–2^ and only slightly decreased after 50 cycles. Meanwhile, the cyclability of the MoS_2_@CF‐NRGO configuration at 4.2 mg cm^–2^ was maintained after 200 cycles at 1 C (Figure S33, Supporting Information). This demonstrates the excellent synergistic effect of MoS_2_ strongly coupled with the 3D carbon matrix to effectively alleviate the shuttle effect in high‐areal‐capacity Li–S batteries. Subsequently, soft‐pack batteries with MoS_2_@CF‐NRGO/PP were assembled and could charge a mobile phone even in the folding state (Figure 4i).

**Figure 5:**
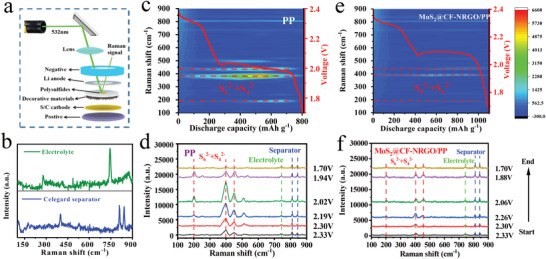
In situ micro‐Raman measurements of the MoS_2_@CF‐NRGO/PP and PP separators during discharge: a) Schematic illustration of Li‐S configurations with a sealed glass window for in situ Raman experiments. b) Raman spectra of the PP separator and Li‐S electrolyte. c,e) In situ time‐resolved Raman images of the PP and MoS_2_@CF‐NRGO/PP separators, respectively. d,f) Selected Raman spectroscopy of the PP and MoS_2_@CF‐NRGO/PP separators, respectively. The inset red curves in (c) and (e) are voltage profiles of the PP and MoS_2_@CF‐NRGO/PP separators, respectively.

To better understand the shuttling effect of LiPSs, their evolution was analyzed by in situ Raman spectroscopy at the Li anode side during the discharging process (**Figure** **5**). Figure 5a shows the construction diagram of the device, in which LiPSs can be qualitatively detected in real time using a quartz observation window. Figure 5b presents the Raman spectra of the PP separator and electrolyte used to eliminate redundant Raman peaks during the testing process. As clearly presented in Figure 5c,d, the characteristic peaks of long‐chain and mid‐chain polysulfides were observed in the Raman image and Raman spectra in the PP configuration.^[^ ^45^ ^]^ In particular, at the low‐voltage plateau, the peak intensity of polysulfides in the PP configuration was clearly heightened because of the penetration of massive polysulfides from the sulfur cathode to the Li anode. With continuous discharge, the polysulfide signal gradually weakened, which indicates that the polysulfides abscised from the PP separator and diffused to the lithium anode. These results demonstrate that a significant shuttle effect occurred in the PP configuration. Conversely, the in situ Raman spectra of the MoS_2_@CF‐NRGO/PP configuration are shown in Figure 5e,f, in which the peak intensity of the polysulfides was weak during the all‐discharge process, indicating only a small amount of polysulfide shuttling. This further confirms that MoS_2_@CF‐NRGO enables stronger adsorption and effective inhibition of intermediate polysulfides.

## 3 Conclusions

DFT calculations revealed that the intrinsic electronic conductivity of MoS_2_ and the smooth electronic pathway of the heterogeneous interface of MoS_2_/C can significantly accelerate bidirectional solid–liquid–solid conversion. Then, MoS_2_@CF‐NRGO with covalent coupling of the Mo–O–C bond, as a multifunctional modified layer, was prepared for Li–S batteries. A series of experiments, such as anchoring/catalytic analyses and in situ Raman spectroscopy, proved that this functional separator can regulate Li_2_S deposition and facilitate Li_2_S decomposition. Li–S batteries with an MoS_2_@CF‐NRGO‐modified separator presented low self‐discharge (only 8.6%), excellent rate performance (615 mAh g^−1^ at 5 C), stable cycling performance (after 1000 cycles at 1 C with a capacity decay rate of only 0.06% per cycle), and a high areal capacity (6.11 mAh cm^−2^ at 0.2 C). Composites with strong covalent coupling can be extended to other materials, facilitating the development of composite electrocatalysts for high‐performance Li–S batteries.

## 4 Experimental Section

Experimental details were shown in the Supporting Information.

## Conflict of Interest

The authors declare no conflict of interest.

## Supporting information

Supporting Information

## Data Availability

The data that support the findings of this study are available from the corresponding author upon reasonable request.

## References

[1] Y. Liu , Z. Wei , B. Zhong , H. Wang , L. Xia , T. Zhang , X. Duan , D. Jia , Y. Zhou , X. Huang , Energy Storage Mater. 2021, 35, 12.

[2] S. Huang , Z. Wang , Y. Lim , Y. Wang , Y. Li , D. Zhang , H. Yang , Adv. Energy Mater. 2021, 11, 2003689.

[3] B. Fei , C. Zhang , D. Cai , J. Zheng , Q. Chen , Y. Xie , L. Zhu , A. Cabot , H. Zhang , ACS Nano 2021, 15, 6849. 33769793 10.1021/acsnano.0c10603

[4] H. J. Peng , J. Q. Huang , X. B. Cheng , Q. Zhang , Adv. Energy Mater. 2017, 7, 1700260.

[5] K. Xu , X. Liu , J. Liang , J. Cai , K. Zhang , Y. Liu , X. Wu , M. Zhu , Y. Liu , Y. Zhu , ACS Energy Lett. 2018, 3, 420.

[6] G. Zhou , S. Zhao , T. Wang , S. Z. Yang , B. Johannessen , H. Chen , C. Liu , Y. Ye , Y. Wu , Y. Peng , Nano Lett. 2020, 20, 1252. 31887051 10.1021/acs.nanolett.9b04719

[7] B. Zhang , J. Wu , J. Gu , S. Li , T. Yan , X. P. Gao , ACS Energy Lett. 2021, 6, 537.

[8] Z. Li , Q. Zhang , L. Hencz , J. Liu , P. Kaghazchi , J. Han , L. Wang , S. Zhang , Nano Energy 2021, 89, 106331.

[9] M. Chen , X. Zhao , Y. Li , P. Zeng , H. Liu , H. Yu , M. Wu , Z. Li , D. Shao , C. Miao , Chem. Eng. J. 2020, 385, 123905.

[10] J. Y. Hwang , H. M. Kim , S. K. Lee , J. H. Lee , A. A. Abouimrane , M. A. Khaleel , I. Belharouak , A. Manthiram , Y. K. Sun , Adv. Energy Mater. 2016, 6, 1501480.

[11] J. Balach , J. Jaumann , M. Klose , S. Oswald , J. Eckert , L. Giebeler , Adv. Funct. Mater. 2015, 25, 5285.

[12] Z. L. Xu , J. K. Kim , K. Kang , Nano Today 2018, 19, 84.

[13] W. Bao , L. Liu , C. Wang , S. Choi , D. Wang , G. Wang , Adv. Energy Mater. 2018, 8, 1702485.

[14] L. Luo , Z. Qin , J. Wu , G. Liang , Q. Li , M. Liu , F. Kang , G. Chen , B. Li , J. Mater. Chem. A 2018, 6, 8612.

[15] Q. Li , Z. Ma , J. Zhao , K. Shen , T. Shi , Y. Xie , Y. Fan , X. Qin , G. Shao , J. Power Sources 2022, 521, 230929.

[16] Q. Li , Z. Ma , J. Li , Z. Liu , L. Fan , X. Qin , G. Shao , ACS Appl. Mater. Interfaces 2020, 12, 35049. 32667773 10.1021/acsami.0c09583

[17] B. Moorthy , S. Kwon , J. H. Kim , P. Ragupathy , H. M. Lee , D. K. Kim , Nanoscale Horiz. 2019, 4, 214. 32254159 10.1039/c8nh00172c

[18] H. Liu , H. Cheng , H. Jin , C. Gao , P. Zhang , M. Wang , Adv. Mater. 2021, 2, 688.

[19] Q. Hu , J. Lu , C. Yang , C. Zhang , J. Hu , S. Chang , H. Dong , C. Wu , Y. Hong , L. Zhang , Small 2020, 16, 2002046. 10.1002/smll.202002046 32697433

[20] Z. Cheng , Y. Chen , Y. Yang , L. Zhang , H. Pan , X. Fan , S. Xiang , Z. Zhang , Adv. Energy Mater. 2021, 11, 2003718.

[21] D. Tian , X. Song , Y. Qiu , X. Sun , B. Jiang , C. Zhao , Y. Zhang , X. Xu , L. Fan , N. Zhang , ACS Nano 2021, 15, 16515. 34590820 10.1021/acsnano.1c06067

[22] H. Lin , S. Zhang , T. Zhang , H. Ye , Q. Yao , G. Zheng , J. Y. Lee , Adv. Energy Mater. 2019, 9, 1902096.

[23] J. Yang , L. Yu , B. Zheng , N. Li , J. Xi , X. Qiu , Adv. Sci. 2020, 7, 1903260. 10.1002/advs.201903260 PMC7610341 33173722

[24] J. Wu , X. Li , H. Zeng , Y. Xue , F. Chen , Z. Xue , Y. Ye , X. Xie , J. Mater. Chem. A 2019, 7, 7897.

[25] B. Chen , T. Wang , S. Zhao , J. Tan , N. Zhao , S. Jiang , Q. Zhang , G. Zhou , H. Cheng , Adv. Mater. 2021, 33, 2007090. 10.1002/adma.202007090 33599013

[26] H. J. Yoo , S. S. Mahapatra , J. W. Cho , J. Phys. Chem. C 2014, 118, 10408.

[27] Z. Qiao , Y. Zhang , Z. Meng , Q. Xie , L. Lin , H. Zheng , B. Sa , J. Lin , L. Wang , D. L. Peng , Adv. Funct. Mater. 2021, 31, 2100970.

[28] H. Wang , D. Wei , J. Zheng , B. Zhang , M. Ling , Y. Hou , C. Liang , ACS Appl. Energy Mater. 2020, 3, 11893.

[29] D. Kong , H. Wang , J. J. Cha , M. Pasta , K. J. Koski , J. Yao , Y. Cui , Nano Lett. 2013, 13, 1341. 23387444 10.1021/nl400258t

[30] M. B. Sreedhara , A. L. Santhosha , A. J. Bhattacharyya , C. N. R. Rao , J. Mater. Chem. A 2016, 4, 9466.

[31] M. Liu , C. Zhang , J. Su , X. Chen , T. Ma , T. Huang , A. Yu , ACS Appl. Mater. Interfaces 2019, 11, 20788. 31074966 10.1021/acsami.9b03011

[32] Q. Fan , J. Jiang , S. Zhang , T. Zhou , W. K. Pang , Q. Gu , H. Liu , Z. Guo , J. Wang , Adv. Energy Mater. 2021, 11, 2100957.

[33] Y. Dai , X. Liao , R. Yu , J. Li , J. Li , S. Tan , P. He , Q. An , Q. Wei , L. Chen , X. Hong , K. Zhao , Y. Ren , J. Wu , Y. Zhao , L. Mai , Adv. Mater. 2021, 33, 2100359. 10.1002/adma.202100359 33998711

[34] P. Li , H. Lv , Z. Li , X. Meng , Z. Lin , R. Wang , X. Li , Adv. Mater. 2021, 33, 2007803. 10.1002/adma.202007803 33734507

[35] S. Jiang , M. Chen , X. Wang , Z. Wu , P. Zeng , C. Huang , Y. Wang , ACS Sustainable Chem. Eng. 2018, 6, 16828.

[36] H. Lin , L. Yang , X. Jiang , G. Li , T. Zhang , Q. Yao , G. Zheng , J. Y. Lee , Energy Environ. Sci. 2017,10, 1476.

[37] F. Y. Fan , W. C. Carter , Y. M. Chiang , Adv. Mater. 2015, 27, 5203. 26257297 10.1002/adma.201501559

[38] Z. Yuan , H. J. Peng , T. Z. Hou , J. Q. Huang , C. M. Chen , D. W. Wang , X. B. Cheng , F. Wei , Q. Zhang , Nano Lett. 2016, 16, 519. 26713782 10.1021/acs.nanolett.5b04166

[39] X. Zhang , Y. Wei , B. Wang , M. Wang , Y. Zhang , Q. Wang , H. Wu , Nano‐Micro Lett. 2019, 11, 78. 10.1007/s40820-019-0313-x PMC7770752 34138023

[40] K. Chen , G. Zhang , L. Xiao , P. Li , W. Li , Q. Xu , J. Xu , Small Methods 2021, 5, 2001056. 10.1002/smtd.202001056 34927835

[41] Y. Yi , Z. Liu , P. Yang , T. Wang , X. Zhao , H. Huang , Y. Cheng , J. Zhang , M. Li , J. Energy Chem. 2020, 45, 18.

[42] S. D. Seo , D. Park , S. Park , D. W. Kim , Adv. Funct. Mater. 2019, 29, 1903712.

[43] Z. Li , L. Tang , X. Liu , T. Song , Q. Xu , H. Liu , Y. Wang , Electrochim. Acta 2019, 310, 1.

[44] X. Li , A. Lushington , Q. Sun , W. Xiao , J. Liu , B. Wang , Y. Ye , K. Nie , Y. Hu , Q. Xiao , R. Li , J. Guo , T. K. Sham , X. Sun , Nano Lett. 2016, 16, 3545. 27175936 10.1021/acs.nanolett.6b00577

[45] W. Chen , T. Lei , T. Qian , W. Lv , W. He , C. Wu , X. Liu , J. Liu , B. Chen , C. Yan , J. Xiong , Adv. Energy Mater. 2018, 8, 1702889.

