# Supplementary material for: Mo‐O‐C Between MoS2 and Graphene Toward Accelerated Polysulfide Catalytic Conversion for Advanced Lithium‐Sulfur Batteries
Source: Adv Sci (Weinh). 2022 Jun 5;9(22):2201579. doi: 10.1002/advs.202201579 (PMC9353409; doi:10.1002/advs.202201579)
Supplement: Supplementary file 1 — Supporting Information [file ADVS-9-2201579-s001.pdf]

ADVANCED SCIENCE
Open Access

Supporting Information

for *Adv. Sci.*, DOI: 10.1002/advs.202201579

Mo-O-C Between MoS2 and Graphene Toward Accelerated Polysulfide Catalytic Conversion for Advanced Lithium-Sulfur Batteries

Jiayu Zhang, Guobao Xu, Qi Zhang,* Xue Li, Yi Yang, Liwen Yang, Jianyu Huang, and Guangmin Zhou**

Mo-O-C between MoS_2 and Graphene towards Accelerated Polysulfide Catalytic Conversion for Advanced Lithium-Sulfur Batteries

Jiayu Zhang[a], *Guobao Xu*[a*], *Qi Zhang*[a,b*], *Xue Li*[a], *Yi Yang*[a], *Liwen Yang*[c], *Jianyu Huang*[a] , *Guangmin Zhou*[b*]

[a] School of Materials Science and Engineering, Xiangtan University, 411105, Hunan, China

[b] Shenzhen Geim Graphene Center, Tsinghua-Berkeley Shenzhen Institute & Tsinghua Shenzhen International Graduate School, Tsinghua University, Shenzhen 518055, China

[c] School of Physics and Optoelectronics, Xiangtan University, Hunan 411105, China

[*]Corresponding authors: gbxu@xtu.edu.cn(G.B.Xu); qizhang91@sz.tsinghua.edu.cn (Q.Zhang); guangminzhou@sz.tsinghua.edu.cn

Preparation of CF-rGO precursor

4 g ammonium bicarbonate was dissolved in 75 mL deionized water, and then 300 mg graphene oxide was added and dispersed by sonication. The melamine foam was rinsed with alcohol to remove some contaminants and dried at 60 ℃ for 2 h. The dried melamine foam put into the above graphene oxide aqueous solution to soak and then dried through freeze-dry. Subsequently, the dried foam was calcined at 600 ℃ for 2 h with a heating rate of 5 ℃ min^{-1} under pure Ar flow. After that, the 3D porous nitrogen doped reduction of graphene oxide supported on carbon foam (CF-rGO) precursor was obtained.

Preparation of MoS_2@CF-rGO

To fabricate the MoS_2@CF-rGO, a piece of as-prepared CF-rGO precursor was first immersed in a teflon-lined stainless-steel autoclave containing a mixed solution with 60 mg thiourea and 30 mg sodium molybdate, which kept at 200 ℃ for 24 h. After the hydrothermal treatment, the sample was soaked in deionized water to remove the residual impurities and dried through freeze-dry to obtain the MoS_2@CF-rGO precursor. Finally, the precursor was annealed at 600 ℃ for 2 h under Ar gas to get MoS_2@CF-rGO. Meanwhile, for comparison, the NRGO supported on graphitic carbon foam (CF-rGO) was obtained by annealing CF-rGO precursor at 600 ℃ for 2 h with a heating rate of 5 ℃ min^{-1} under pure Ar flow. In additional, in order to prove that nanoflower-like MoS_2 has abundant edge site, the 60 mg thiourea and 30 mg sodium molybdate was dissolved in 30 mL deionized water, and then put into a teflon-lined stainless-steel autoclave and keep at 200 ℃ for 24 h. After MoS_2 microsphere was obtained by further annealed at 600 ℃ for 2 h under Ar gas, as shown in **Fig S10**, mark as pure MoS_2. Meanwhile, the pure MoS_2 and CF were mixed in deionized water and dried to

obtain a physically mixed compounds (marked as MoS_2-CF-NRGO), thus to demonstrate the enhancement of Mo-O-C band on the catalytic activity of the material.

Fabrication of function separator

The obtained MoS_2@CF-rGO or CF-rGO and PVDF (9:1 by mass) were uniformly dispersed in N-methylpyrrolidone (NMP) by sonication for 30 min. The mixed solution was further coated on a polypropylene (PP) separator (Celgard 2325) by using a vacuum filtration method, and then vacuum dried at 60 °C for 12 h. Finally, the obtained modified separator was punched into disks with a diameter of 19 mm for the separator of lithium-sulfur batteries.

Adsorption tests of polysulfides

The Li_2S_6 with a concentration of 3 mmol L^{-1} were prepared by mixing S powder and Li_2S at molar ratio of 5:1 in a mixed solvent 1,2-dimethoxyethane (DME) and 1,3-dioxolane (DOL) (v/v=1:1) under magnetic stirring for 48 h. 10 mg of MoS_2@CF-rGO and CF-rGO was introduced into the Li_2S_6 solution (5 mL), respectively, followed by aging for 24 h.

Fabrication of Li_2S_6 symmetric cells and measurements

Functional materials (MoS_2@CF-rGO or CF-rGO) and PVDF binder with a weight ratio of 9:1 was uniformly mixed into the NMP and then coated onto the aluminum foil. After dried at 60 °C for 12 h and punched into disks with a diameter of 12.0 mm, the areal loading of the obtained electrode was about 1 mg cm^{-2}. Then, the identical working and counter electrodes of MoS_2@CF-rGO, CF-rGO with a PP separator, and 40 μL electrolyte containing 0.5 M Li_2S_6 in a mixed solvent DME and DOL (v/v=1:1) were fabricated into the symmetric cells. CV tests were carried out with a CHI660E electrochemical working station. The CV curves were recorded at a scan rate of 10 mV s^{-1} with in the voltage range of -1.5 -1.5 V.

Nucleation and dissolution of Li_2S test

A Li_2S_8 solution (0.5 mol L^{-1}) were prepared by mixing S powder and Li_2S at molar ratio of 7:1 in tetraglyme solution under magnetic stirring for 48 h. Similarly, 12 mm diameter aluminum foil coated with functional materials (MoS_2@CF-rGO or CF-rGO) and PVDF binder with a weight ratio of 9:1 was used as working electrode to assemble the cell and a lithium foil was used as the anode. During the cell assembly process, 10 μL Li_2S_8 (0.5 mol L^{-1}) was dropped onto the cathode and then 10 μL Li-S battery electrolyte (1.0 mol L^{-1} of bis(triuoromethane) sulfonimide lithium (LiTFSI) and 0.2 mol L^{-1} lithium nitrate ($LiNO_3$) dissolved in DOL/DME (v/v = 1/1)) was dropped on the lithium anode side. The cell was galvanostatically discharged to 2.06 V under the current of 0.112 mA, and the potential was then kept at 2.05 V until the current dropped below 10^{-5} A. Driven by an overpotential of 0.01 V, Li_2S was deposited and it grew on the cathode surface. The cells of dissolution test are exactly the same as that of the nucleation test. The assembled cells were firstly galvanostatically discharged to 1.8 V at 0.1 mA for complete transform of Li_2S_8 to solid Li_2S, and subsequently the cells were potentiostatically charged at 2.4 V for the oxidization of Li_2S into soluble polysulfides.

Li–Li symmetric cells

The electrodes were prepared by Li metal foils with diameter of 12 mm, the PP separator and MoS_2@CF-rGO, CF-rGO modified separator were used as separators, and using 40 μL Li-S battery electrolyte, texted at 2 mA cm^{-2}.

Assembly of Lithium-sulfur batteries and electrochemical measurements

Sulfur cathode was prepared via mixing sublimed sulfur, acetylene black and PVDF binder with a weight ratio of 6:3:1 was mixed in NMP to form a homogeneous slurry. The slurry was coated onto aluminum foil, followed by drying at 60 °C for 12 h in a vacuum oven and punched into discs with a diameter of 12 mm, the sulfur loading is about 1-1.2 mg cm^{-2}. Sulfur/carbon cathode was prepared via mixing sublimed sulfur and acetylene black with a weight ratio of 8:2. Subsequently, the uniformly mixed powder was heated at 155 °C for 12 h in Ar-filled autoclave. The obtained S/C composite mixing acetylene black and PVDF binder with a weight ratio of 8:1:1 was mixed in NMP to form a homogeneous slurry. The slurry was coated onto carbon cloth (WOS1009), followed by drying at 60 °C for 12 h in a vacuum oven and punched into discs with a diameter of 12 mm, the areal sulfur loading is about 2-10 mg cm^{-2}. **Figure 21 a-b** displayed the SEM images of S and S/C composite cathodes and corresponding EDS of sulfur. **Figure 21 c-d** shown that the XRD spectrums of S and S/C composite cathodes. The cross-sectional SEM images of S and S/C composite cathodes are shown in **Figure S21e-f**.

After that, standard coin cell (CR2025) with S or S/C cathode, Li anode, and functional separator was assembled in Ar-filled glove box. The electrolyte was consisted of 1.0 mol L^{-1} of bis(triuoromethane) sulfonimide lithium (LiTFSI) and 0.2 mol L^{-1} lithium nitrate ($LiNO_3$) dissolved in DOL/DME (v/v =1/1). The electrolyte/sulfur ratios are around 30 uL mg^{-1} for the cathodes with 1-1.2 mg cm^{-2} sulfur loading, 10 uL mg^{-1} for the cathodes with 2.0-10 mg cm^{-2} sulfur loading. The galvanostatic charge/discharge performance tests and the rate capability at different C-rates were performed using a Neware battery test system. Cyclic voltammetry (CV) measurements and electrochemical impedance spectroscopy (EIS) were carried out using a

CHI660E electrochemical working station. The EIS was measured in the frequency range of $0.01\text{-}10^5$ Hz.

In situ Raman spectroscopy

SIBs/Li-S batteries with a quartz window, provided by Beijing Science Star Technology Co. Ltd, were used for in situ micro-Raman spectroscopy analysis. For SIBs, the electrode slurry was pasted onto nickel foam after stirring and tailored to a disk after being heated in a vacuum overnight. The conditions for assembling the battery are consistent with the button batteries. For Li-S batteries, the same method was used to prepare cathodes as button batteries. A hole was created on lithium metal foil to allow the laser shed on the separator. The cells were run at a (dis)charging rate of 50 mA g^{-1} (SIBs) and 0.5 C (LSBs). Raman signals were recorded simultaneously by a 532 nm laser.

Calculation method

All the calculations were performed based on the density functional theory used the Perdew-Burke-Ernzerh exchange-correlational functional of generalized gradient approximation,[1] and the projector-augmented wave method,[2] implemented by Vienna Ab-initio Simulation Package (VASP).[3] The cutoff energy for the plane wave-basis expansion was set to 500 eV and the atomic relaxation was continued until both the force acting on atoms was smaller than 0.01 eV $Å^{-1}$ and the energy was converged to 1×10^{-5} eV at the same time.

The Gibbs free energy change (ΔG) of each lithiation step was defined as: $\Delta G = \Delta E + \Delta ZPE - T\Delta S$, where ΔE is the electronic energy difference directly obtained from DFT calculations, ΔZPE is the change in zero-point energy, T is the temperature (T= 298.15 K) and

ΔS is the change in the entropy, respectively. The zero-point energy and entropy were obtained through vibrational frequencies.

The dissociation energy barrier of Li_2S^* was calculated with the support of the climbing image-nudged elastic band (CI-NEB) method.[4]

Characterization

X-ray powder diffraction profiles (XRD) of the obtained samples were recorded on a Rigaku D/MAX 2500. The Raman scattering patterns were obtained from Renishaw InVia system. The composition of the products was characterized by X-ray photoelectron spectroscopy (Kratos Analytical Ltd., UK) using an Al Kα source. The morphology, microstructure, and composition of the obtained materials were carried out by scanning electron microscopy (SEM, Hitachi, S4800) and transmission electron microscope (TEM, JEOL JEM-2010, 200 kV).

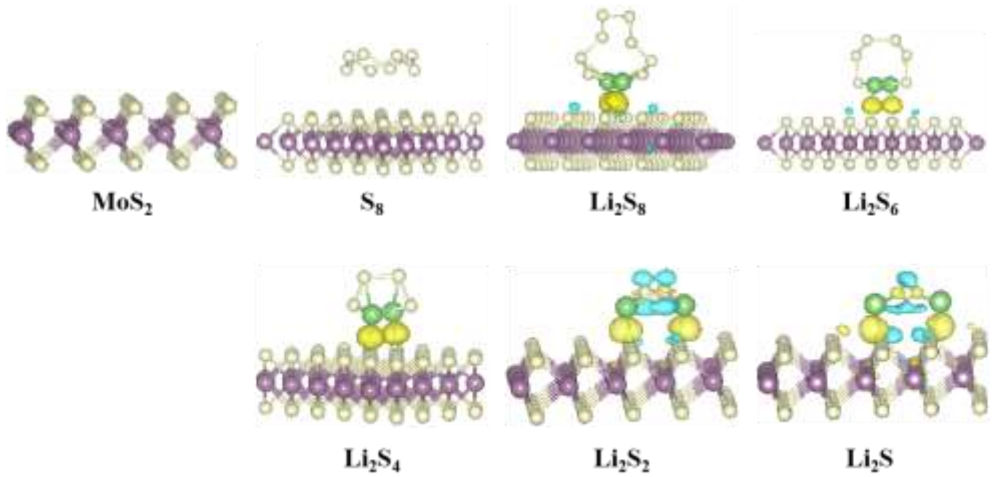

Figure S1. DFT-calculated molecular structures of pure MoS_2, and optimized configurations for the binding of Li_2Sn and S_8 to the pure MoS_2.

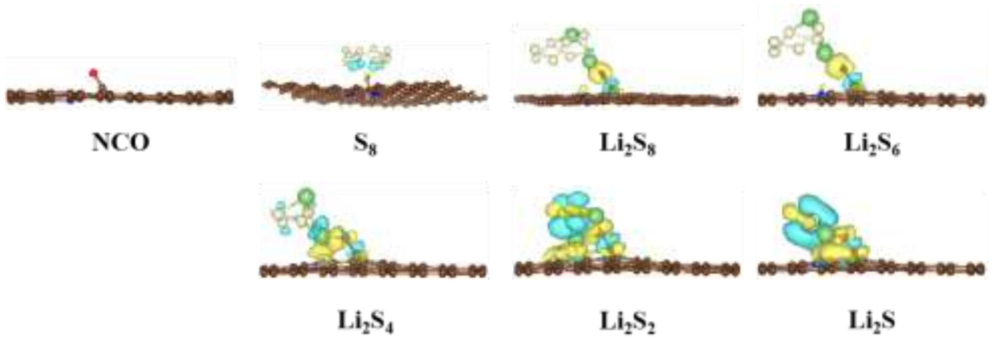

Figure S2. DFT-calculated molecular structures of pure NCO, and optimized configurations for the binding of Li_2Sn and S_8 to the NCO.

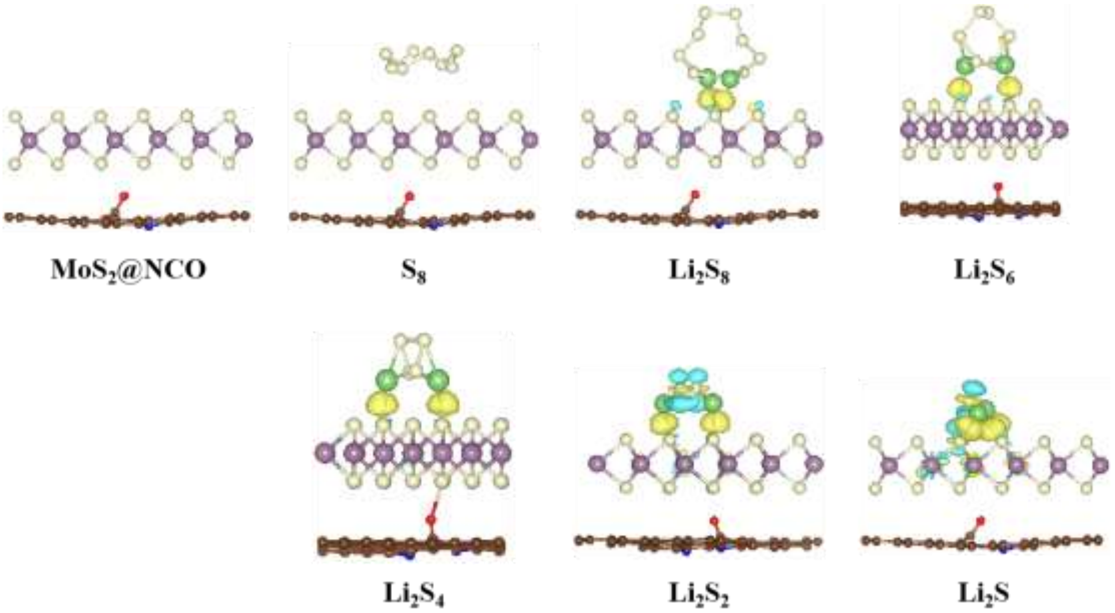

Figure S3. DFT-calculated molecular structures of MoS_2@NCO, and optimized configurations for the binding of Li_2Sn and S_8 to the MoS_2@NCO.

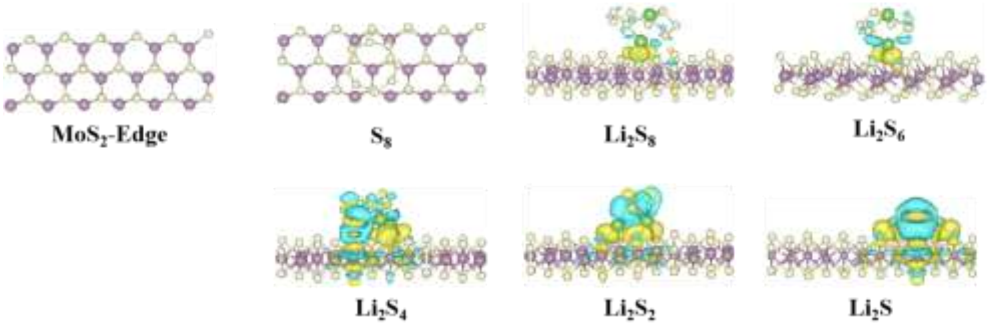

Figure S4. DFT-calculated molecular structures of MoS_2-Edge, and optimized configurations for the binding of Li_2Sn and S_8 to the MoS_2-Edge.

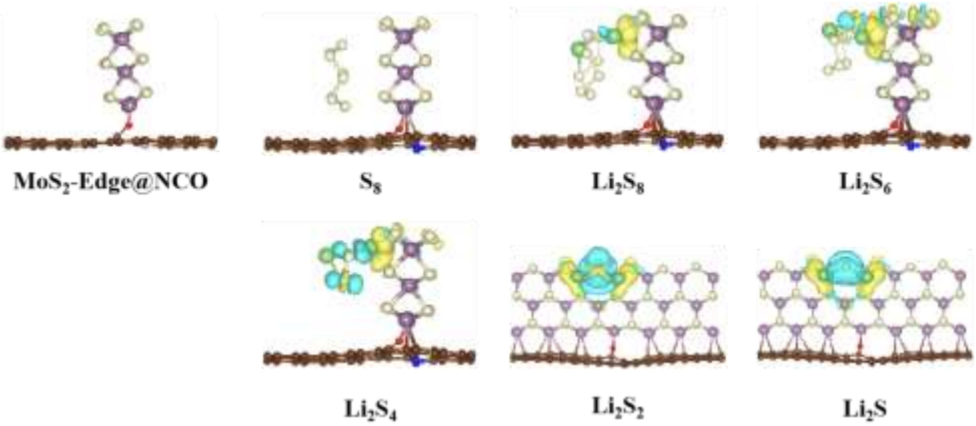

Figure S5. DFT-calculated molecular structures of MoS$_2$-Edge@NCO, and optimized configurations for the binding of Li$_2$Sn and S$_8$ to the MoS$_2$-Edge@NCO.

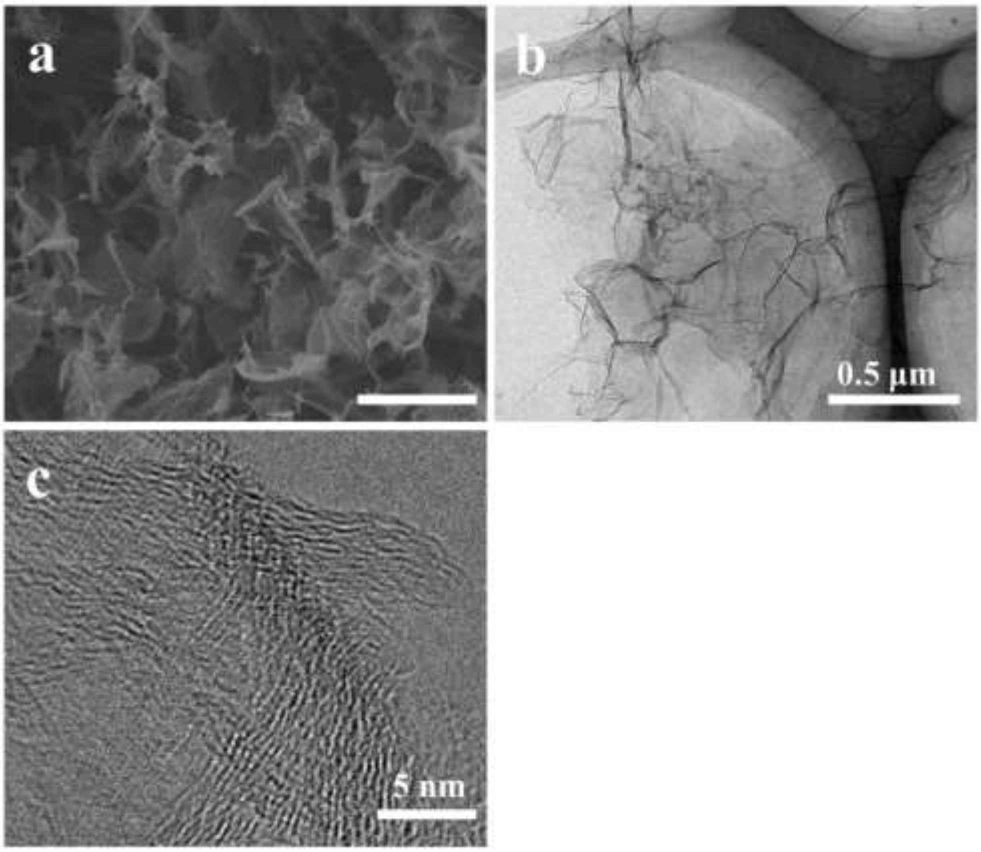

Figure S6. a) SEM image, b) TEM and c) HR-TEM images of CF-NRGO

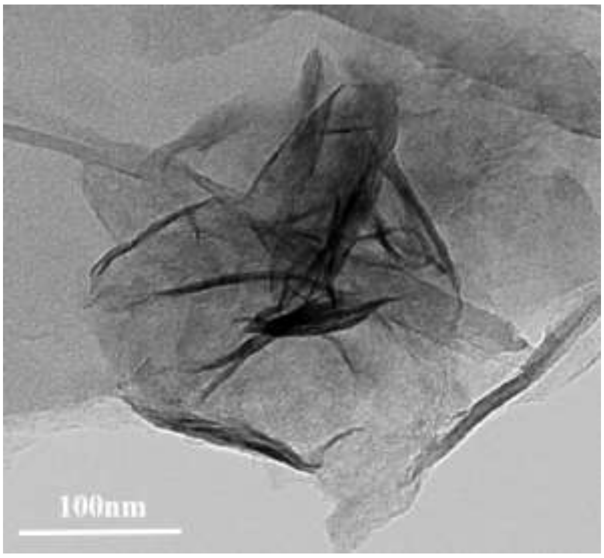

Figure S7. TEM image of MoS_2@CF-NRGO

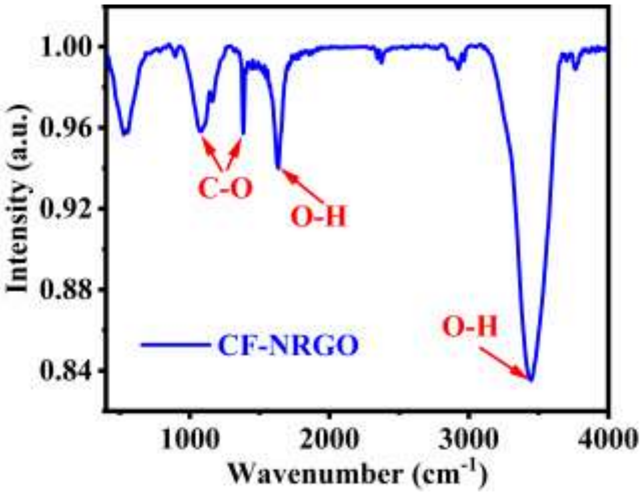

Figure S8. FTIR spectra of the CF-NRGO

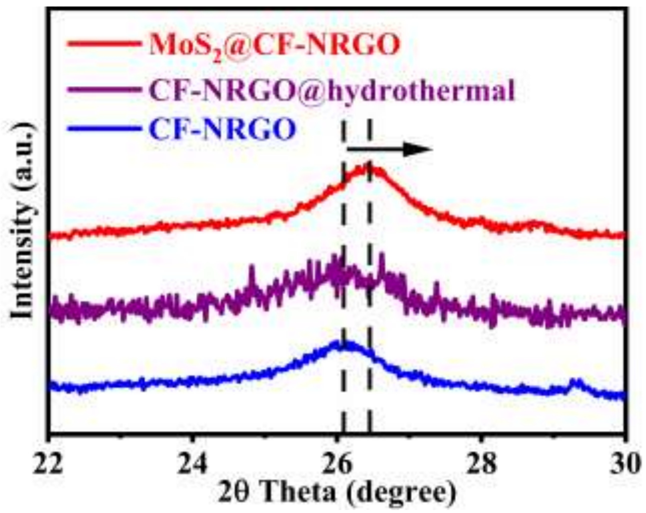

Figure S9. The detailed comparison of XRD diffraction peaks at about 26º of MoS_2@CF-NRGO, CF-NRGO and CF-NRGO@hydrothermal.

In order to prove that hydrothermal treatment has no effect on the position of XRD peak at about 26º, a piece of as-prepared CF-NRGO precursor was immersed in a teflon-lined stainless-steel autoclave only containing deionized water, which kept at 200 ℃ for 24 h, the obtained materials mark as CF-NRGO@hydrothermal. The result shows no obvious change of diffraction peak position on the pattern of CF-NRGO@hydrothermal compared with CF-NRGO, indicating that slight increase in lattice distortion great probability derive from the strong interaction between MoS_2 and CF-NRGO.

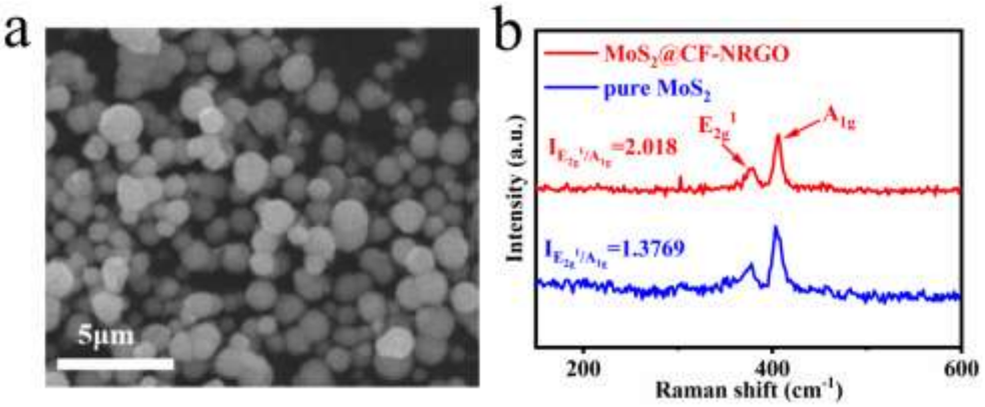

Figure S10. a) SEM image of pure MoS_2 and b) the comparison of Raman spectrum of pure MoS_2 and MoS_2@CF-NRGO.

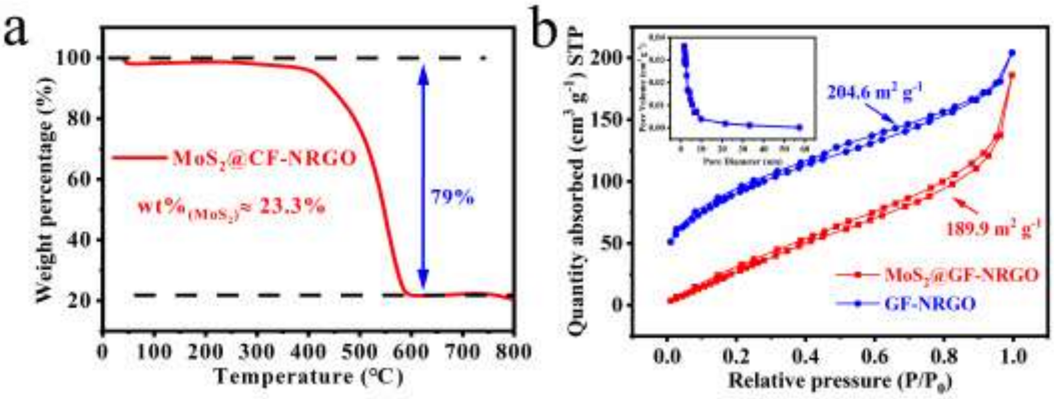

Figure S11. a) TGA curve of MoS_2@CF-NRG in air (the final product was MoO_3). b) N_2 adsorption and desorption isotherms (inset: calculated pore size distribution) of MoS_2@CF-NRGO and CF-NRGO.

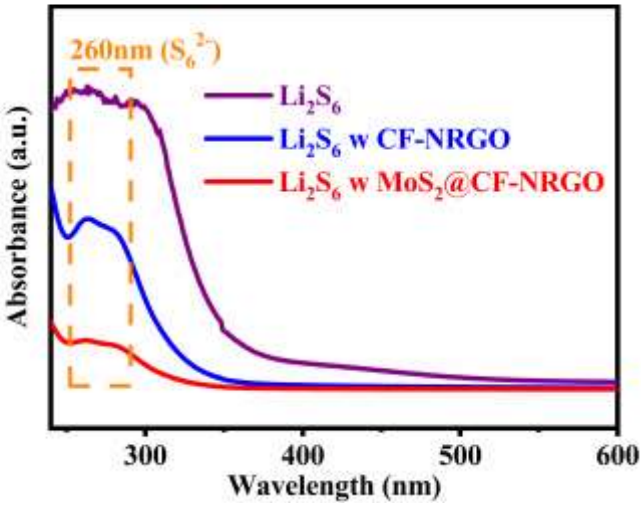

Figure S12. UV-vis absorption spectra of Li_2S_6 solution after exposure to MoS_2@CF-rGO and CF-rGO.

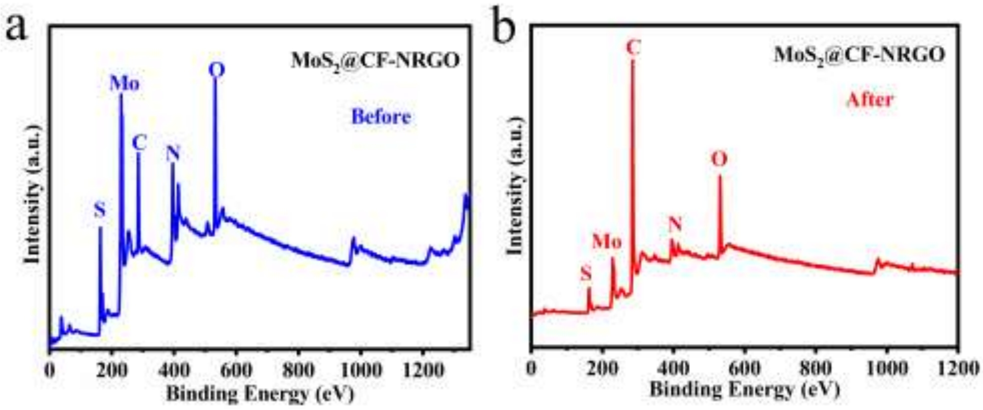

Figure S13. X-ray photoelectron spectroscopy (XPS) of the a) pristine MoS_2@CF-rGO hybrids and b) the adsorbed Li_2S_6.

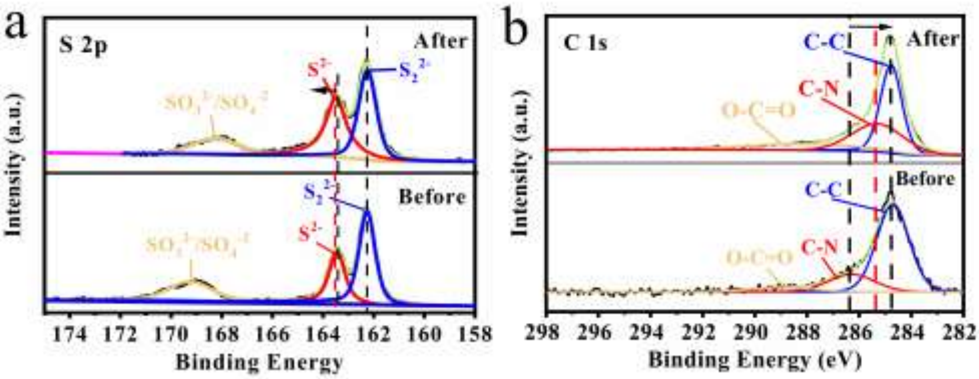

Figure S14. XPS spectra for a) S 2p, b) C 1s of the MoS_2@CF-NRGO before and after adsorbed with Li_2S_6.

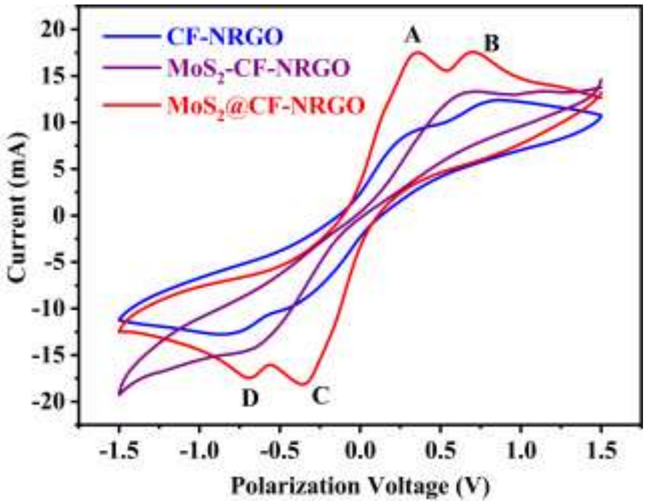

Figure S15. CV curves of symmetric cells of MoS_2@CF-NRGO, CF-NRGO and MoS_2-CF-NRGO electrodes.

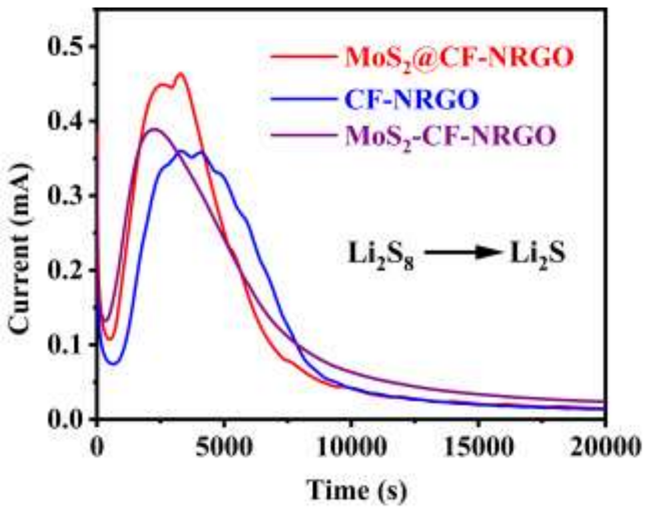

Figure S16. Chronoamperometry discharge curves at 2.05 V of a Li_2S_8/tetraglyme solution on different the surfaces of MoS_2@CF-NRGO, CF-NRGO and MoS_2-CF-NRGO electrodes.

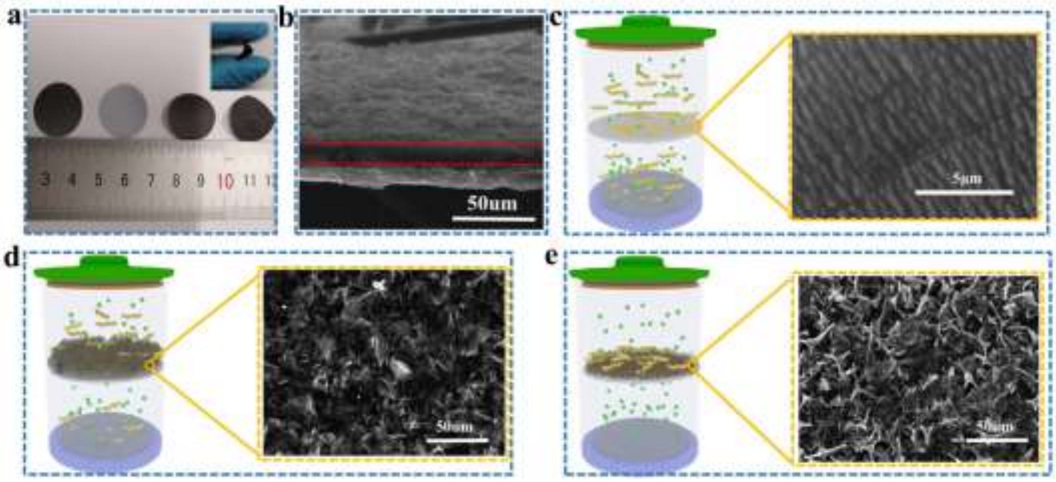

Figure S17. a) Photographs of the front and back sides, flexibility and folding/recovery test of MoS_2@CF-NRGO/PP separator. b) Cross sectional SEM image of MoS_2@CF-NRGO/PP separator. SEM images and schematic diagram in Li-S batteries of c) PP separator, d) CF-NRGO/PP, and e) MoS_2@CF-NRGO/PP separator.

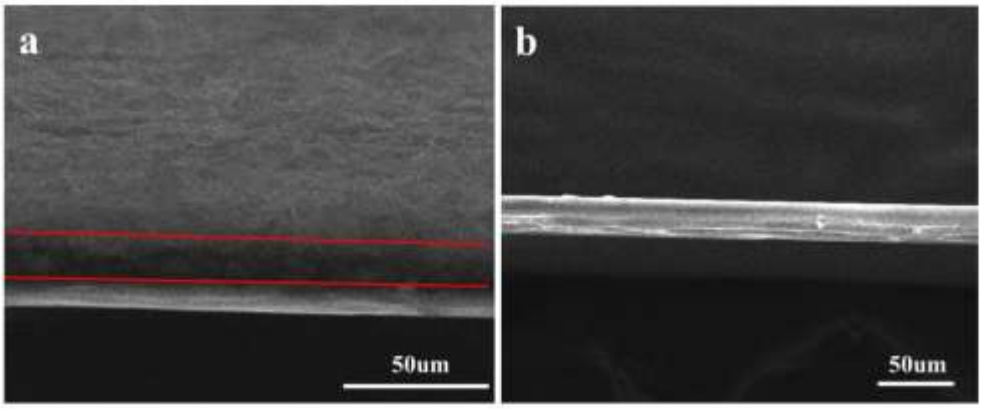

Figure S18. Cross section SEM image of a) CF-NRGO/PP and b) PP separator

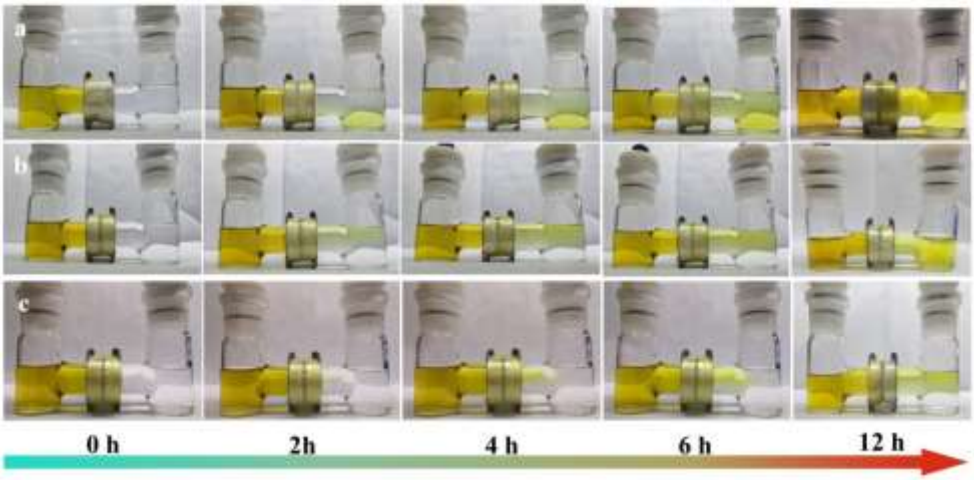

Figure S19. Polysulfide (3 mM Li_2S_6) permeation test using H-type glass cells with a) pristine PP separator, b) CF-NRGO/PP separator, and c) MoS_2@CF-NRGO/PP separator.

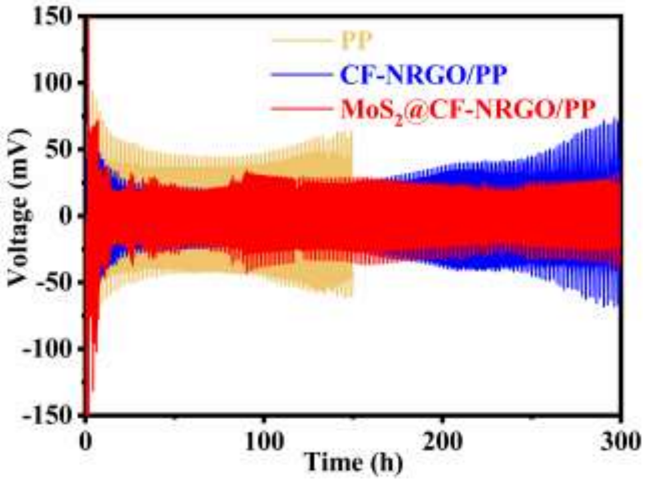

Figure S20. Li-Li symmetric cells with MoS_2@CF-NRGO/PP separator, CF-NRGO/PP separator, and PP separator.

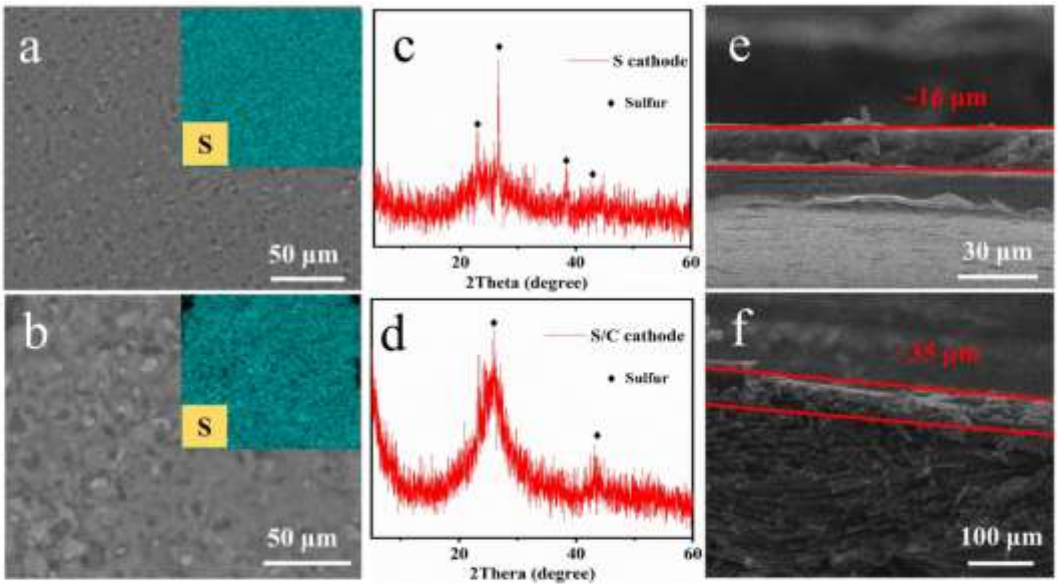

Figure S21. The SEM images of a) S and b) S/C composite cathodes, insect: corresponding EDS mapping of sulfur distribution. The XRD spectrums of c) S and d) S/C composite cathodes. The cross-sectional SEM images of e) S and f) S/C composite cathodes.

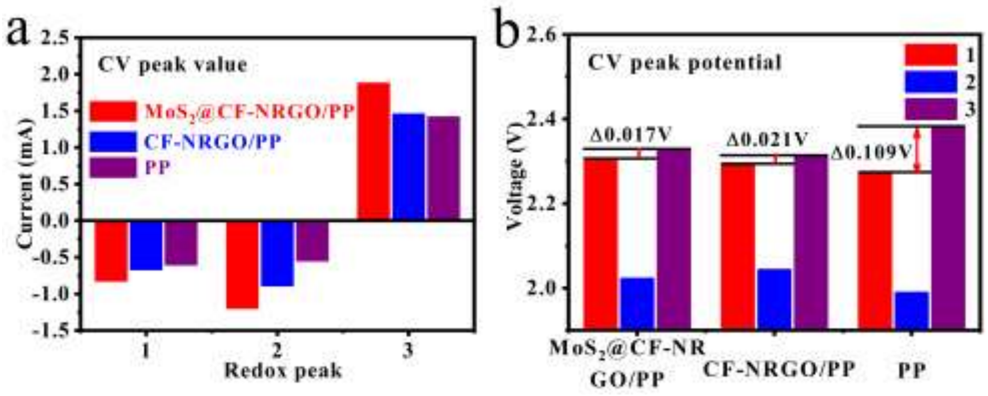

Figure S22. Corresponding a) peak current and b) peak voltages and polarization voltage of the cell with MoS_2@CF-NRGO/PP, CF-NRGO/PP and PP separators.

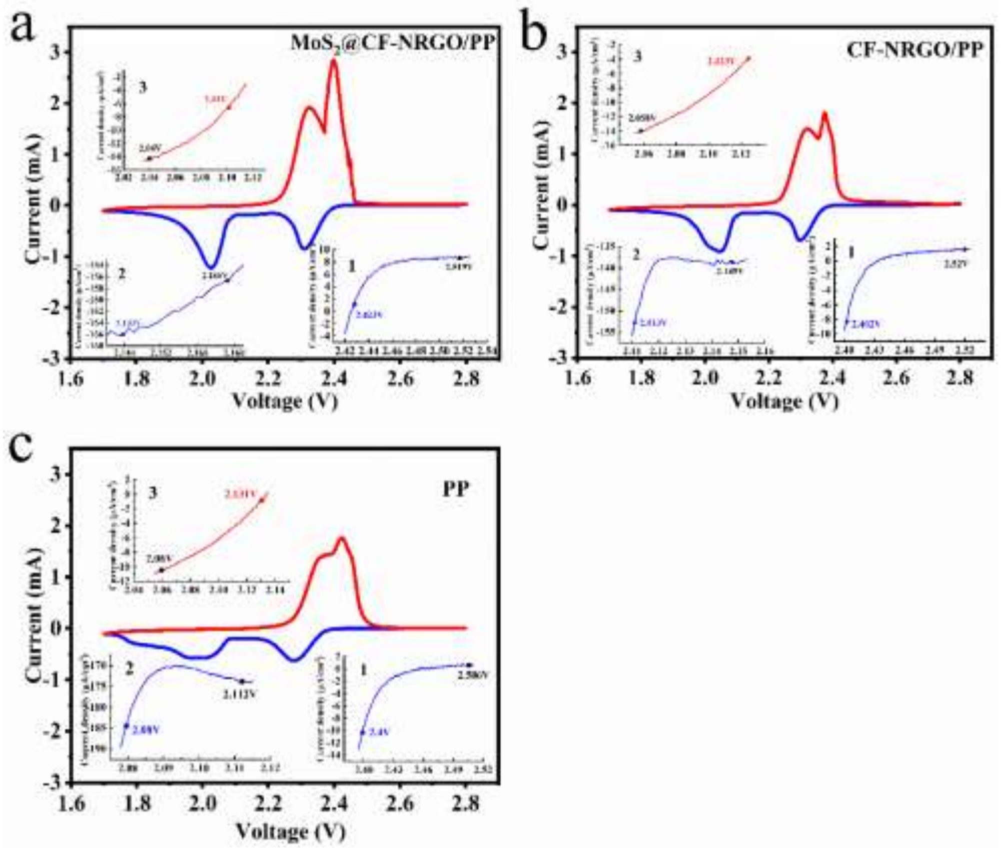

Figure S23. CV curves of cell with a) MoS_2@CF-NRGO/PP separator, b) CF-NRGO/PP separator, and c) PP separator at scan rate of 0.1mV s^{-1}, and their corresponding onset potentials of redox peaks provided as the insets.

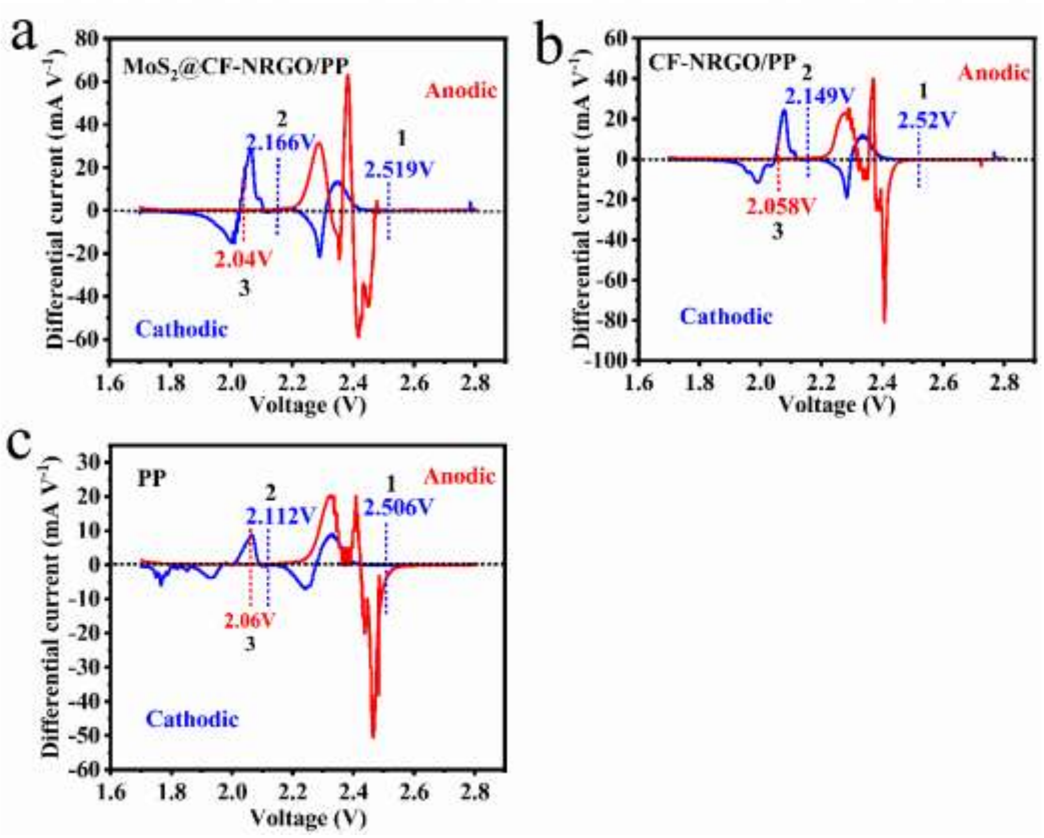

Figure S24. a, b, c) differential CV curves of MoS_2@CF-NRGO, CF-NRGO and PP separators. The baseline potentials and baseline current densities in (a, b, c) are defined as the values before the redox peaks, where the variation on current density is the smallest, namely dI/dV=0. Baseline voltages are denoted in blue for cathodic peak 1, 2 and in red for anodic peak 3, respectively.

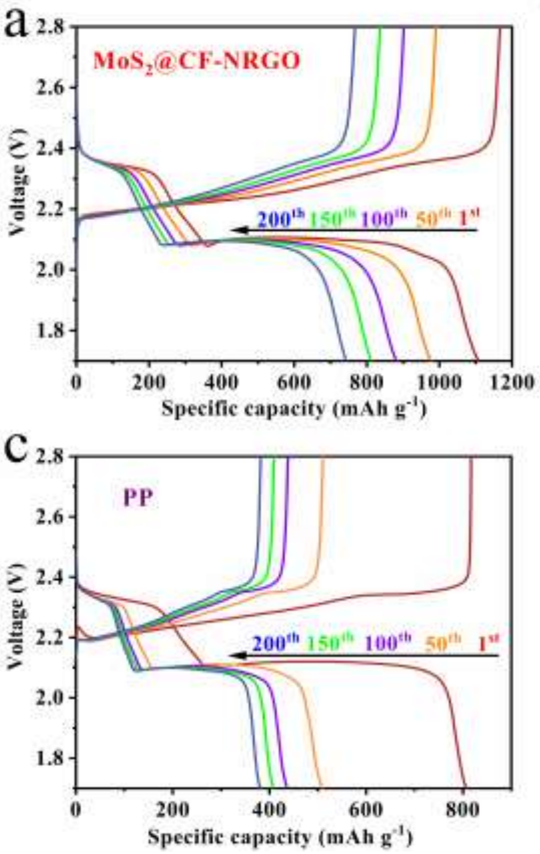

Figure S25. Galvanostatic discharge/charge profiles of a) MoS_2@CF-NRGO, b) CF-NRGO, and c) PP separators at 0.2 C current rate

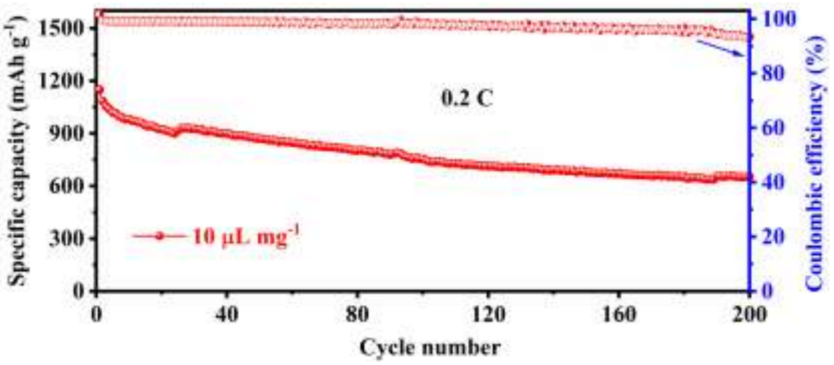

Figure S26. Cycling performance at 0.2 C of MoS_2@CF-NRGO/PP separator with electrode/sulfur ratio of 10 μL mg^{-1}.

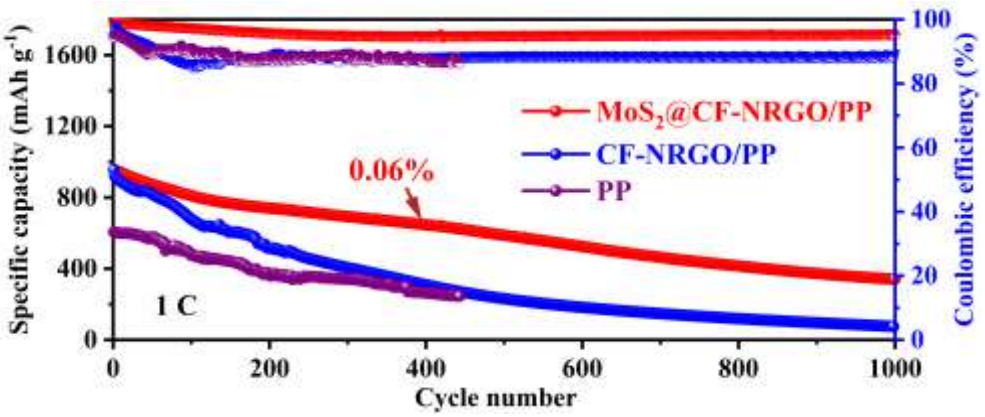

Figure S27. cycling stability at 1 C for different separators.

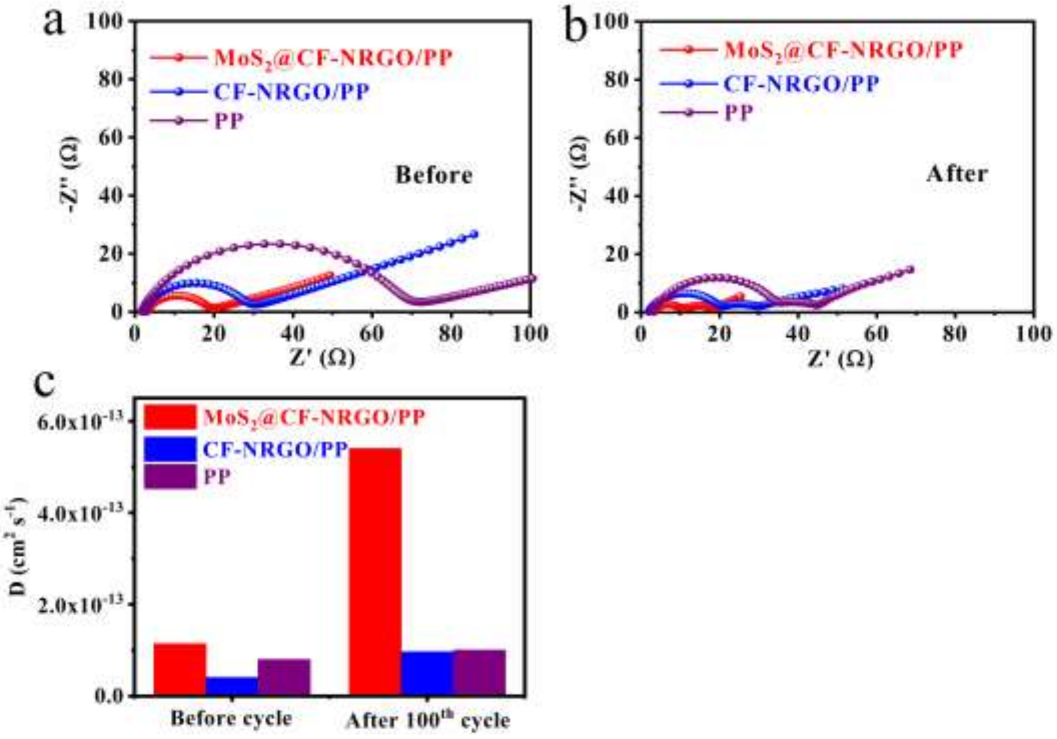

Figure S28. The Nyquist plots of the EIS spectra and c) calculated D (cm^2 s^{-1}) of the Li-S cells with different separator a) before cycling and b) after 200 cycles at 1 C.

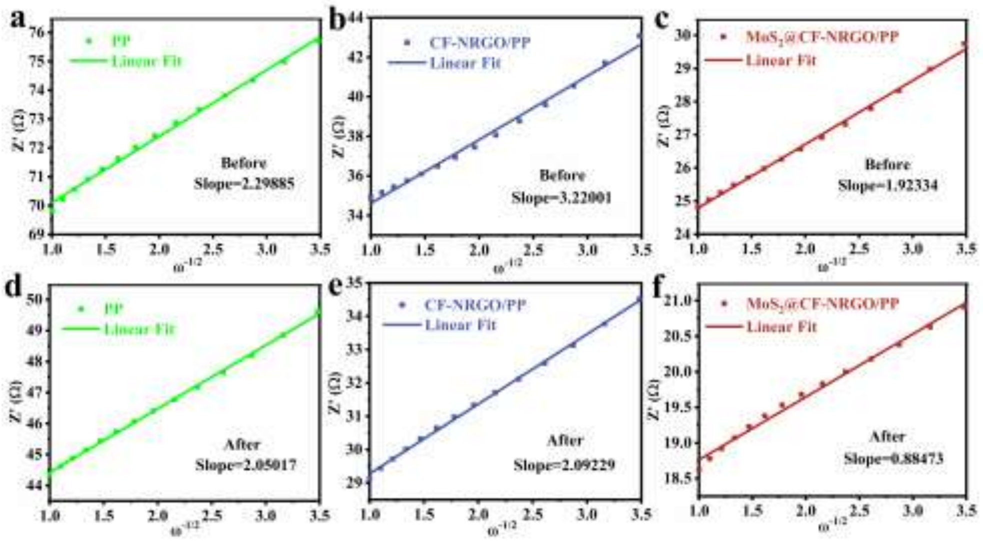

Figure S29. Relationship between Z′ and $\omega^{-1/2}$ in the middle -frequency region (a-c) before cycling and (d-f) after 100 cycles.

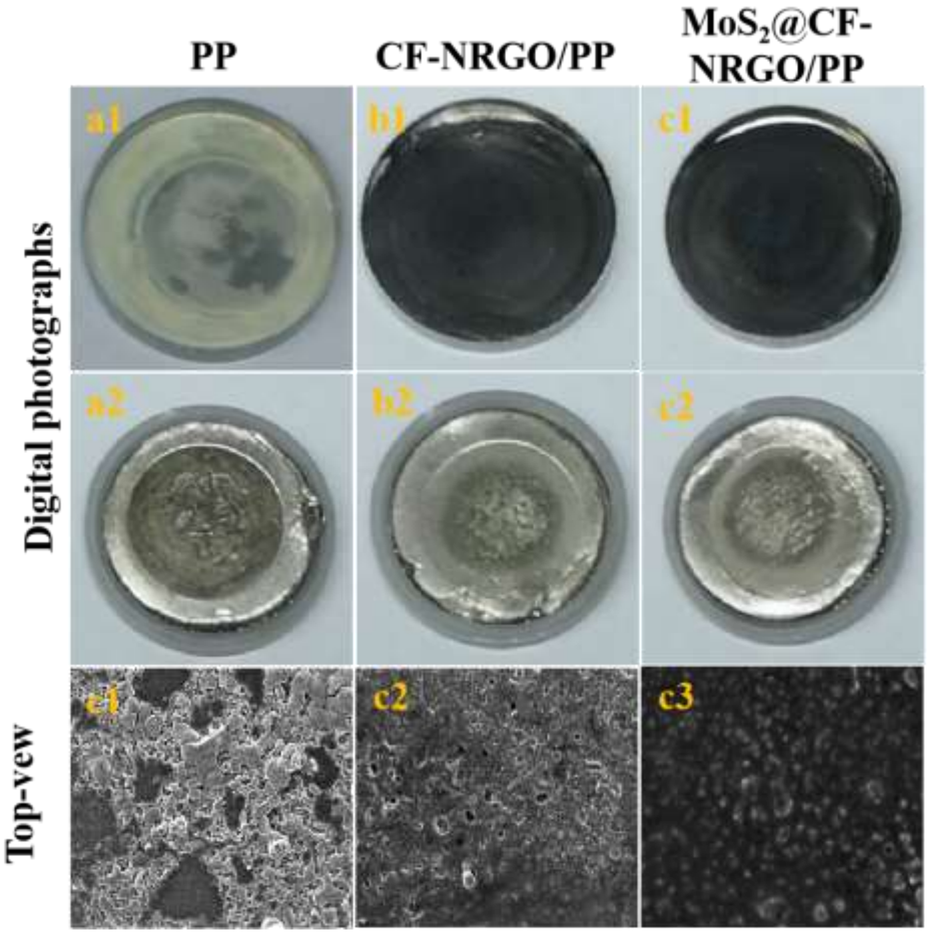

Figure S30 Digital photographs and SEM images (Top-view) of different separators after testing at 1 C for 200 cycles.

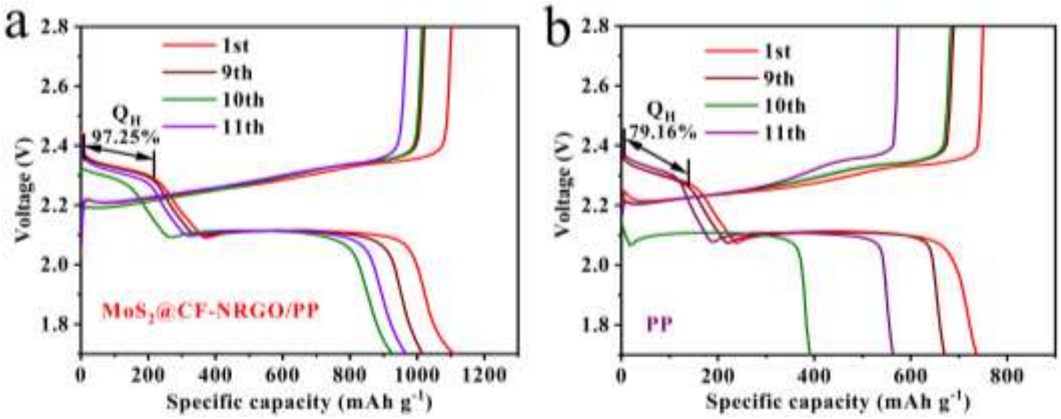

Figure S31. Galvanostatic charge/discharge curves of the cells with a) MoS_2@CF-NRGO/PP separator, and b) PP separator at 0.5 C before and after rest.

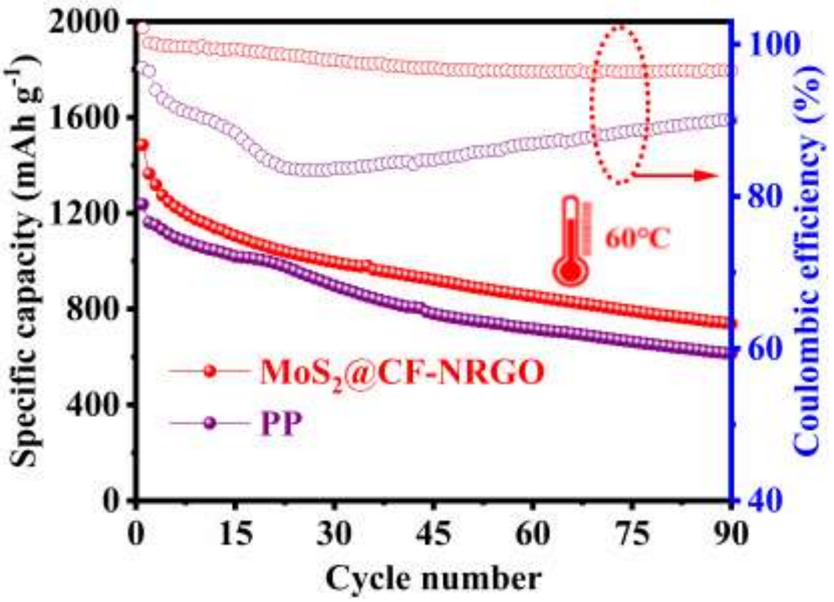

Figure S32. Cycling performance at 0.2 C of MoS_2@CF-NRGO/PP and PP separators at 60 ℃

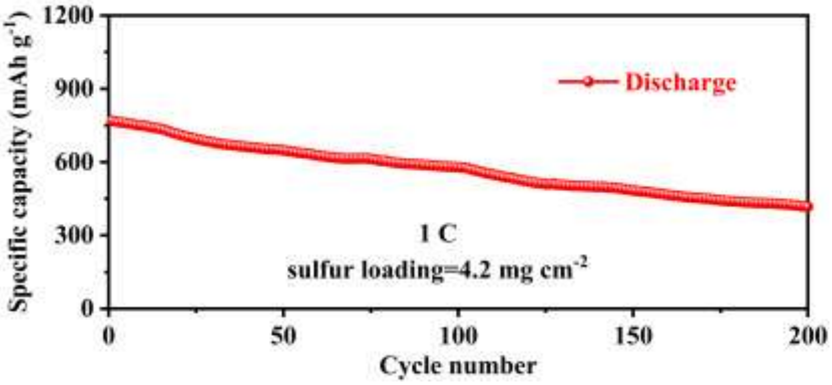

Figure S33. Cycling performance at 1 C of the MoS_2@CF-NRGO/PP separator.

Table S1 Comparison of electrochemical performance of the Li-S batteries some reported recently.

Separator materials	Method	Mass loading of layer (mg/cm^2)	Sulfur materials	Preparation method of sulfur materials	Sulfur loading (mg/cm^2)	Initial capacity (mAh/g)/ Rate (C)	Rate (C)/ Decay rate (% per cycle) /Cycle number	Rate properties (mAh/g)/Rate (C)	Areal capacity (mAh/cm^2)/sulfur loading (mg/cm^2)/Rate (C)	Ref
MoS_2@CF-NRGO	Separator Modification	0.3	S	\	~1	1120/0.2C	1C/0.064/1000	615.1/5C	6.11/8.0/0.2C	This work
MoS_2	Separator Modification	\	S	\	\	1039/0.2C	0.5C/0.083/600	550/1C	\	[5]
CF@2H/1T MoS_2	Separator Modification	4.1	S	\	1.1-1.5	1510/0.2C	2C/0.0492/1000	501/5C	3.9/5.7/0.2C	[6]
rGO-QD MoS_2	Host Material	\	\	Melt-diffusion	1.3-1.5	849/0.2C	1C/0.051/1000	293/5C	\	[7]
1T-MoS_2 nanoflowers-graphene	Separator and Host	0.3	\	Melt-diffusion	~1	1245/0.2C	1C/0.058/1000	823/5C	8.54/5.1/1C	[8]
MoS_2/Graphene Microsphere	Separator Modification	0.8	S/Graphene	Ball milling and Melt-diffusion	\	1391/0.2C	0.2C/0.482/100	395/5C	\	[9]
Carbon Clothe@MoS_2	Host Material	\	\	Melt-diffusion	1	0.5C/1086	\	639/2C	\	[10]
MXene/1T-2H MoS_2	Host Material	\	\	Melt-diffusion	2-4	0.1C/1194	0.5C/0.07/300	677.2/2C	\	[11]
Defect-rich MoS_2/N-Doped CNT	Separator Modification	0.48	S	\	1.8-2.0	0.2C/1258	1C/0.11/300	650/2C	\	[12]
MoS_2@SnO_2	Host Material	\	\	Melt-diffusion	2	0.2C/1281	1C/0.033/1000	874/3C	\	[13]
MoS_2-MoN	Host Material	\	\	Solution method	1.2	0.2C/1100	1C/0.039/1000	674/6C	13.3/6.3/0.5C	[14]

P-$Mo_{0.9}CO_{0.1}S_2$	Host Material	\	\	Melt-diffusion	2	0.2C/1485	1C/0.046/600	931/6C	\	[15]
MgO-carbon Foam@CNT	Host Material	\	\	Solution method	1.2-1.5	0.2C/971	2C/0.06/800	753/2C	5.6364/6.6/0.1C	[16]
Porous carbon/Sn_4P_3	Separator Modification	0.1-0.2	MWCNT/S	Melt-diffusion	1-1.2	0.2C/856.2	1C/0.046/900	667.8/3C	5.6/7.1/0.1C	[17]
Fe/Fe_3C@GNC	Separator Modification	1.7	Nano Sulfur	\	1.3	0.2C/1159	1C/0.077/350	725/3C	3.15/3.4/0.2C	[18]
$NiMoO_4$@N-S doped carbon clothes	Host Material	/	/	Solution method and Melt-diffusion	2	0.2C/1156	1C/0.0081/500	691.1/1C	2.8/5/1C	[19]

Table S2 Comparison of self-discharge performances of the Li-S batteries reported recently.

Material	Method	Sulfur loading (mg/cm^2)	Rate (C)	Rest time	Capacity loss rate (%)	Ref
MoS_2@CF-NRGO	Separator Modification	~1	0.5	5 days	8.65	This work
PAN/GO	Separator Modification	0.7-1	0.2	24 h	~27	[20]
ZnS/NCNS	Separator Modification	~1.5	0.5	4 days	4.1	[21]
TiO/MWCNT	Separator Modification	1.4-1.6	0.5	48 h	8.7	[22]
MOCNT-1500	Separator Modification	1.4-1.7	0.5	24 h	5.28	[23]
N-CNTs/Co-NFs	Host Material	3.1	0.2	24 h	5.84	[24]
$FePO_4$/C	Host Material	2	0.2	48 h	22	[25]
C/S/C sandwich structure	Host Material	1-1.4	0.2	3 weeks	~10	[26]

REFERENCES

[1] J. P. Perdew, K. Burke, M. Ernzerhof, *Phys. Rev. Lett.* **1996**, 77, 3865.

[2] P. E. Blöchl, *Phys. Rev. B* **1994**, 50, 17953.

[3] G. Kresse, J. Furthmüller, *Phys. Rev. B* **1996**, 54, 11169.

[4] G. Henkelman, B. P. Uberuaga, H. Jonsson, *J. Chem. Phys.* **2000**, 113, 9901.

[5] Z. A. Ghazi, X. He, A. M. Khattak, N. A. Khan, B. Liang, A. Lqbal, J. Wang, H. Sin, L. Li, Z. Tang, *Adv. Mater.* **2017**, 29, 1606817.

[6] C. Tian, B. Li, X. Hu, J. Wu, P. Li, X. Xiang, X. Zu, S. Li, *ACS Appl. Mater. Interfaces* **2021**, 13, 5, 6229.

[7] H. Wei, Y. Ding, H. Li, Q. Zhang, N. Hu, L. Wei, Z. Yang, *Electrochim. Acta* **2019**, 327, 134994.

[8] Z. Cheng, Y. Chen, Y. Yang, L. Zhang, H. Pan, X. Fan, S. Xiang, Z. Zhang, *Adv. Energy Mater.* **2021**, 11, 2003718.

[9] S. Yang, J. Zhang, T. Tan, Y. Zhao, N. Liu, H. Li, *Materials* **2018**, 11, 2064.

[10] C. Tian, J. Wu, Z. Ma, B. Li, X. Zhang, X. Zu, X. Xiang, S. Li, *Energy Reports* **2020**, 6, 172.

[11] Y. Zhang, Z. Mu, C. Yang, Z. Xu, S. Zhang, X. Zhang, Y. Li, J. Lai, Z. Sun, Y. Yang, Y. Chao, C. Li, X. Ge, W. Yang, S. Guo, *Adv. Funct. Mater.* **2018**, 28, 1707578.

[12] M. Zhen, S. Guo, B. Shen, *ACS Sustainable Chem. Eng.* **2020**, 8, 13318.

[13] F. Li, G. Hu, S. Li, C. Hou, J. Gao, *Electrochim. Acta* **2020**, 340, 135991.

[14] S. Wang, S. Feng, J. Liang, Q. Su, F. Zhao, H. Song, M. Zheng, Q. Sun, Z. Song, X. Jia, J. Yang, Y. Li, J. Liao, R. Li, X. Sun, *Adv. Energy Mater.* **2021**, 11, 2003314.

[15] H. Lin, S. Zhang, T. Zhang, H. Ye, Q. Yao, G. Zheng, J. Y. Lee, *Adv. Energy Mater.* **2019**, 9, 1902096.

[16] M. Xiang, H. Wu, H. Liu, J. Huang, Y. Zheng, L. Yang, P. Jing, Y. Zhang, S. Dou, H. Liu, *Adv. Funct. Mater.* **2017**, 27, 1702573.

[17] Z. Ye, Y. Jiang, T. Feng, Z. Wang, L. Li, F. Wu, R. Chen, *Nano Energy* **2020**, 70, 104532.

[18] M. Zhang, Y. Guo, Y. Wei, B. Wang, Y. Zhang, H. Wu, X. Zhou, Y. Zhang, Q. Wang, *J. Mater. Chem. A* **2020**, 8, 18987.

[19] T. Sun, X. Zhao, B. Li, H. Shu, L. Luo, W. Xia, M. Chen, P. Zeng, X. Yang, P. Gao, Y. Pei, X. Wang, *Adv. Funct. Mater.* **2021**, 2101285.

[20] J. Zhu, C. Chen, Y. Lu, J. Zang, M. Jiang, D. Kim, X. Zhang, *Carbon* **2016**, 101, 272.

[21] Z. Li, F. Zhang, L. Tang, Y. Tao, H. Chen, X. Pu, Q. Xu, H. Liu, Y. Wang, Y. Xia, *Chem. Eng. J.* **2020**, 390, 124653.

[22] Z. Li, L. Tang, X. Liu, T. Song, Q. Xu, H. Liu, Y. Wang, *Electrochim. Acta* **2019**, 310, 1.

[23] C. Choi, D. Lee, J. Park, D. Kim, *ACS Sustainable Chem. Eng.* **2020**, 8, 15134.

[24] L. Ma, H. Lin, W. Zhang, P. Zhao, G. Zhu, Y. Hu, R. Chen, Z. Tie, J. Liu, Z. Jin, *Nano Lett.* **2018**, 18, 7949.

[25] Y. Pang, Y. Wen, W. Li, Y. Sun, T. Zhu, Y. Wang, Y. Xia, *J. Mater. Chem. A* **2017**, 5, 17926.

[26] H. Wang, W. Zhang, H. Liu, Z. Guo, *Angew. Chem. Int. Ed.* **2016**, 55, 3992.
